# Phosphonate production by marine microbes: Exploring new sources and potential function

**DOI:** 10.1073/pnas.2113386119

**Published:** 2022-03-07

**Authors:** Marianne Acker, Shane L. Hogle, Paul M. Berube, Thomas Hackl, Allison Coe, Ramunas Stepanauskas, Sallie W. Chisholm, Daniel J. Repeta

**Affiliations:** ^a^Massachusetts Institute of Technology-Woods Hole Oceanographic Institution Joint Program in Oceanography/Applied Ocean Science and Engineering, Woods Hole Oceanographic Institution, Woods Hole, MA 02543;; ^b^Department of Chemistry and Geochemistry, Woods Hole Oceanographic Institution, Woods Hole, MA 02543;; ^c^Department of Civil and Environmental Engineering, Massachusetts Institute of Technology, Cambridge, MA 02139;; ^d^Department of Biology, University of Turku, Turku 20500, Finland;; ^e^Single Cell Genomics Center, Bigelow Laboratory for Ocean Sciences, East Boothbay, ME 04544;; ^f^Department of Biology, Massachusetts Institute of Technology, Cambridge, MA 02139

**Keywords:** phosphonate, *Prochlorococcus*, marine, biogeochemistry, phosphorus

## Abstract

Phosphonates are organophosphorus metabolites with a characteristic C-P bond. They are ubiquitous in the marine environment, their degradation broadly supports ecosystem productivity, and they are key components of the marine phosphorus (P) cycle. However, the microbial producers that sustain the large oceanic inventory of phosphonates as well as the physiological and ecological roles of phosphonates are enigmatic. Here, we show that phosphonate synthesis genes are rare but widely distributed among diverse bacteria and archaea, including *Prochlorococcus* and SAR11, the two major groups of bacteria in the ocean. In addition, we show that *Prochlorococcus* can allocate over 40% of its total cellular P-quota toward phosphonate production. However, we find no evidence that *Prochlorococcus* uses phosphonates for surplus P storage, and nearly all producer genomes lack the genes necessary to degrade and assimilate phosphonates. Instead, we postulate that phosphonates are associated with cell-surface glycoproteins, suggesting that phosphonates mediate ecological interactions between the cell and its surrounding environment. Our findings indicate that the oligotrophic surface ocean phosphonate pool is sustained by a relatively small fraction of the bacterioplankton cells allocating a significant portion of their P quotas toward secondary metabolism and away from growth and reproduction.

## Significance

Phosphonates are a class of phosphorus metabolites characterized by a highly stable C-P bond. Phosphonates accumulate to high concentrations in seawater, fuel a large fraction of marine methane production, and serve as a source of phosphorus to microbes inhabiting nutrient-limited regions of the oligotrophic ocean. Here, we show that 15% of all bacterioplankton in the surface ocean have genes phosphonate synthesis and that most belong to the abundant groups *Prochlorococcus* and SAR11. Genomic and chemical evidence suggests that phosphonates are incorporated into cell-surface phosphonoglycoproteins that may act to mitigate cell mortality by grazing and viral lysis. These results underscore the large global biogeochemical impact of relatively rare but highly expressed traits in numerically abundant groups of marine bacteria.

Phosphorus (P) is an essential nutrient for all life. Phytoplankton and bacterioplankton (bacteria and archaea) in the surface ocean use P primarily for growth, reproduction, and as an energy currency such that almost all cellular P is associated with rapidly cycled compounds, including nucleic acids, ATP, and phospholipids (1). For example, in the North Pacific subtropical gyre, RNA synthesis accounts for over half of phosphate uptake in phytoplankton communities (2). In times of P scarcity, some marine microbes can modulate their cellular P requirements by recycling RNA from ribosomes (3), substituting phospholipids with other non-P lipids (4), or drawing upon internal stores of P accumulated during periods of P abundance (5, 6). The balance between microbial, physiological processes that require P and those that conserve or internally recycle P has significant downstream consequences for ecological stoichiometry and the marine P biogeochemical cycle (7).

Nevertheless, in nutrient-impoverished midocean gyres, microbial demand for P is often so high that environmental phosphate concentrations are consumed to low-nanomolar levels. Under these conditions, up to half of the microbial P demand is met through the uptake and metabolism of P-containing dissolved organic matter (8). Dissolved organic P (DOP) is a complex, poorly characterized mixture of high– and low–molecular weight (HMW and LMW) phosphate and phosphonate esters released via exudation and cell death. Marine DOP consists primarily of monomeric and polymeric phosphate esters originating from well-known primary metabolites (e.g., nucleotides, nucleic acids, phospholipids, phosphoglycans, phosphoproteins, and vitamins) that are synthesized by all marine microbes. The presence of phosphate esters in DOP is easily explained.

**Fig. 1.**
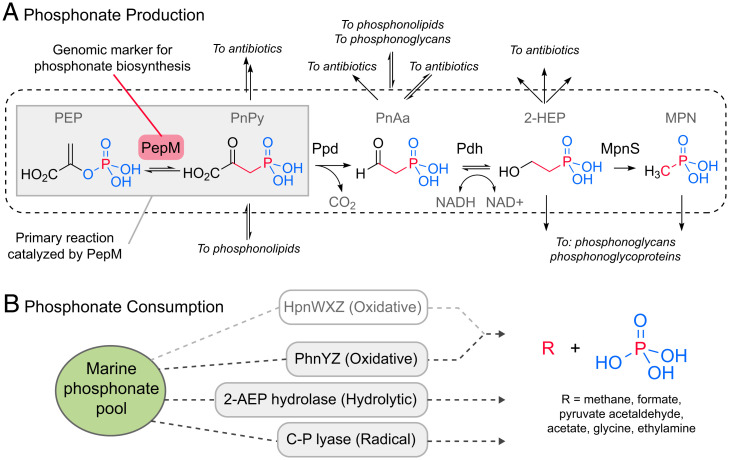
Phosphonate production and consumption in the marine environment. (*A*) This schematic emphasizes reaction pathways (e.g., to methylphosphonate [Mpn]) (18) that are most relevant for planktonic ecosystems in the oligotrophic surface ocean. Branch points to additional natural products are indicated where possible. For a comprehensive overview please refer to ref. 10. The isomerization of phosphoenolpyruvate (PEP) via PEP mutase (PepM, red and gray boxes) produces the phosphonopyruvate (PnPy) precursor from which effectively all known phosphonate compounds are derived. The *pepM* gene is a robust genomic marker for phosphonate production potential due to this functional conservation. Mpn, a well-known substrate for aerobic marine methane production (18), is produced by 1) the decarboxylation of PnPy to phosphonoacetaldehyde (PnAa) via phosphonopyruvate decarboxylase (Ppd), 2) the dehydrogenation of PnAa to 2-hydroxyethylphosphonic acid (2-HEP) via phosphonoacetaldehyde dehydrogenase (Pdh), and finally 3) transformation of 2-HEP by Mpn synthase (MpnS). (*B*) Phosphonate degradation via cleavage of the C-P bond proceeds through at least three mechanisms: hydrolytic (2-AEP, named for the representative 2-aminoethylphosphonate degradation pathway via PhnWX), radical (C-P lyase), and oxidative (PhnYZ). The proposed HpnWXZ pathway (greyed) is likely oxidative but is rare in the genomes we examined.

However, a large fraction of HMWDOP, between 20 and 25%, occurs as phosphonates (9)—an enigmatic class of compounds with a direct carbon to P (C-P) bond—that are not products of the primary cellular metabolism (Fig. 1) (10). Phosphonates were initially believed to be rare and unusual compounds with little environmental relevance (11). However, recent studies have indicated that many marine microbes have the potential to degrade and use phosphonates as a nutritional P source (Fig. 1) (12–14). This has been experimentally confirmed (15–17), showing that degradation of phosphonates in HMWDOP is an important source of marine methane, thereby contributing to the supersaturation of methane in oxic oligotrophic waters (18)—the so-called “marine methane paradox” (19).

Although it is now recognized that phosphonates support a significant fraction of microbial P demand in the ocean, only two bacterioplankton species have been experimentally confirmed as phosphonate producers: *Trichodesmium erythraeum*, a nitrogen-fixing cyanobacterium (20), and *Nitrosopumilus maritimus* from the Marine Group I (MGI) Thaumarchaeota (21). These two groups by themselves can only account for a small fraction of the total inventory of phosphonates observed in surface ocean DOP (*SI Appendix*, *SI Results*) (20).

Most of our understanding of the distribution of the phosphonate synthesis trait in the wild is derived from genomics approaches using phosphonate biosynthesis genes as biomarkers (21–23). The enzyme phosphonenolpyruvate mutase (PepM) is required to form the initial C-P bond in the phosphonopyruvate precursor from which all other known phosphonate biosynthetic products are derived (Fig. 1). This makes the *pepM* gene a key biomarker for phosphonate biosynthetic potential. Metagenomic studies have estimated that 8 to 16% of all surface bacterioplankton may be phosphonate producers, including the abundant marine groups *Prochlorococcus* and SAR11 (21, 22). However, none of these organisms have been experimentally shown to produce phosphonate, and little is known about the functional roles that phosphonates play in *Prochlorococcus* and SAR11 ecology (10).

Here, we first expand on earlier metagenomic studies and incorporate thousands of randomly sampled single-cell genomes from across the tropical and subtropical surface ocean to identify potential phosphonate producers and to quantify their potential contribution to the phosphonate pool. The single-cell genomes allowed us to determine whether individual cells are capable of both producing and consuming phosphonates. The expanded dataset also allowed us to explore the frequencies of phosphonate production and consumption traits among closely related groups and identify associations of these traits with environmental variables. Further, we identified a cultured strain of *Prochlorococcus* that has the full phosphonate biosynthetic pathway and demonstrate that it produces high–molecular weight phosphonate compounds. Finally, we identified the macromolecular cellular components with which the phosphonates are associated giving us clues as to their functional roles in ocean ecosystems.

## Results and Discussion

### Phosphonate-Cycling Genes Are Common in SAR11 and *Prochlorococcus*.

**Fig. 2.**
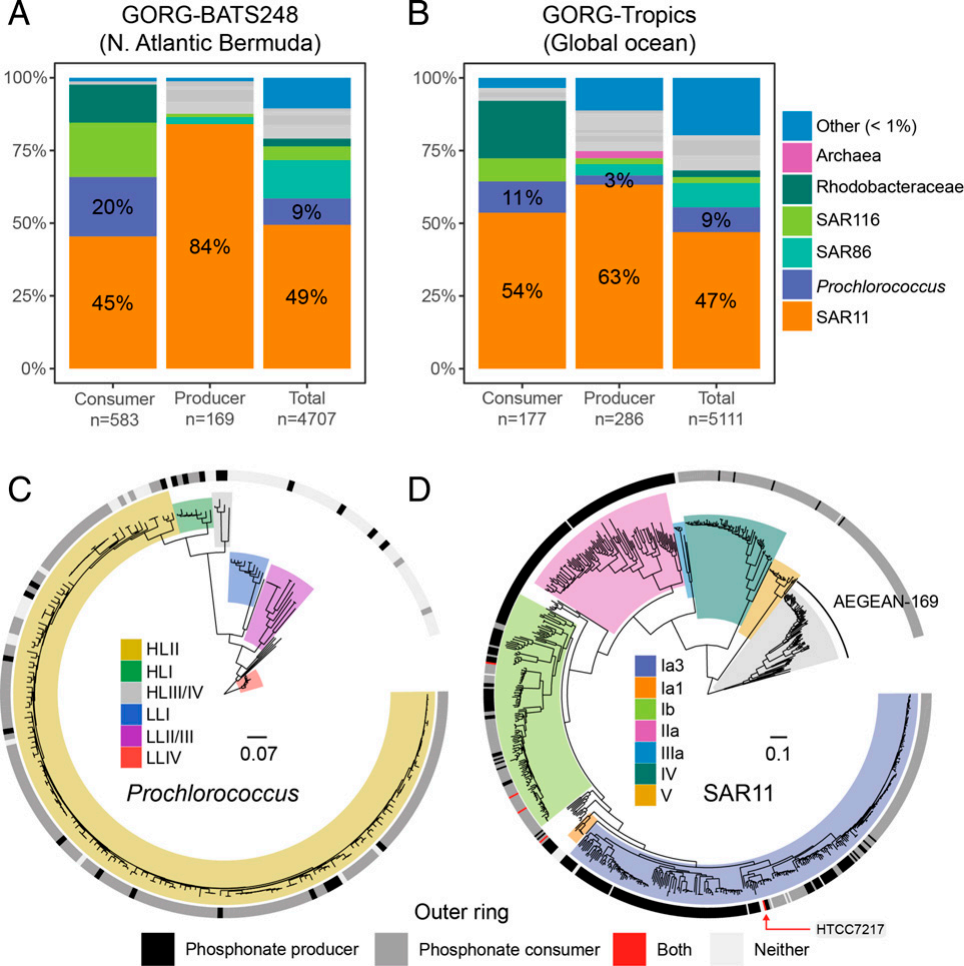
SAR11 and *Prochlorococcus* potential for phosphonate production and consumption in the surface ocean. Taxonomic assignments (as percent of total cells) of phosphonate consumers, producers, and all (total) genomes from the following: (*A*) Bermuda in the North Atlantic Ocean (GORG-BATS248) and (*B*) the global tropical ocean (GORG-Tropics). The number of genomes in each category are listed on the *x*-axis. Taxonomic groups with abundance less than 1% of the total are collapsed. Gray bars represent nonfocal taxonomic groups (*SI Appendix*, Fig. S1). Here, GORG-Tropics contains a random selection of genomes from GORG-BATS248 subsampled to the median per-sample abundance in GORG-Tropics. Genome phylogenies of (*C*) *Prochlorococcus* (n = 235) and (*D*) SAR11 (n = 695) single-cell genomes from GORG-Tropics and isolate genomes. Dominant clades/ecotypes from each group are highlighted. The ring outside each tree displays whether a genome is a phosphonate producer (black), a phosphonate consumer (gray), or both a producer and consumer (red). Isolate genomes with neither pathway are shown in white. For reference, HTCC7217 from the SAR11 clade 1a3 is both a producer and consumer. *Prochlorococcus* is rooted on the LLIV clade and SAR11 is rooted on the AEGEAN-169 group. Scale bars represent amino acid substitution per site.

How widespread is the putative ability to produce and consume phosphonates among diverse marine microbes? To address this question, we used a genome-resolved approach with thousands of randomly sampled single-cell amplified genomes (SAGs) from the Global Ocean Reference Genome (GORG-Tropics) dataset (24), which contained cells from the tropical and subtropical surface ocean. The sequenced cells in this database were randomly selected, thus minimizing bias. Assembly ambiguities are further avoided, because the genomic information is known to come from a single cell. Putative phosphonate producers—as defined by the presence of the *pepM* gene (Fig. 1)—were found in 15% of the bacterioplankton, belonging to diverse taxa, in this dataset (*SI Appendix*, *SI Results* and Fig. S1 and Datasets S1 and S2). A total 9% of the cells in a single deeply sequenced sample from the North Atlantic (GORG-BATS248) were producers (*SI Appendix*, Table S1). The two most-numerically abundant and cosmopolitan marine groups in the tropical surface ocean—SAR11 and *Prochlorococcus*—have the potential to be significant phosphonate producers, as the trait is found in 18% and 6% of their cells, respectively. Together, SAR11 and *Prochlorococcus* account for over half of the cells with this trait in the GORG dataset (Fig. 2 *A* and *B*). SAR11 are small, chemoheterotrophic Alphaproteobacteria estimated to constitute up to half of the total plankton cells in the surface ocean (25), while *Prochlorococcus* numerically dominates unicellular, photoautotrophic picocyanobacteria in the euphotic zone of subtropical and tropical oligotrophic areas (26).

We next asked how abundant phosphonate degradation pathways were in the GORG-Tropics dataset. Approximately 10% of all bacterioplankton in the tropical surface ocean encode genes for phosphonate consumption—defined by the presence of at least one of four distinct degradation pathways (Fig. 1 and *SI Appendix*, Table S1). A total 31% of the cells in GORG-BATS248 (i.e., at a single station in the Atlantic) had the genomic potential to degrade phosphonates, consistent with relatively high North Atlantic consumer abundances inferred from metagenomic studies (27). Overall, however, the proportions of putative producers and consumers are similar across the entire tropical surface ocean (15 and 10%, respectively).

### Phosphonate Production and Consumption Traits Are Rarely Found in the Same Cell.

Both putative phosphonate producers and consumers can be found within closely related taxonomic groups in the GORG dataset (e.g., the *Prochlorococcus* genus contains both). Therefore, we next asked whether phosphonate production and consumption traits were partitioned at the level of clades within these groups or at the level of single cells. Neither production nor consumption traits were correlated with the *Prochlorococcus* phylogeny, indicating that most *Prochlorococcus* clades contain both phosphonate producers and consumers (Fig. 2*C*). In SAR11, the correlation between traits and phylogeny was stronger, particularly for clades IIa, IV, and V—the former one being producers and the latter clades mostly consumers (Fig. 2*D*). However, the highly abundant SAR11 surface clades Ia and Ib contained both producers and consumers in roughly equal proportions. Remarkably, only 0.7% (9/1,234) of all genomes contained both production and consumption traits (i.e., in the SAR11, SAR116, and Roseobacter groups). The presence of both phosphonate consumption and production traits may be advantageous for some bacterial lifestyles (e.g., rare facultative oligotrophs that can also quickly respond to resource pulses). However, the rarity of this trait combination in the wild suggests that it is prohibitively costly for most bacteria.

We also examined tens of thousands of other microbial genomes from marine, freshwater, terrestrial, and host-associated habitats (*Materials and Methods*) to determine whether the pattern of production and consumption exclusivity held over a wider taxonomic and habitat breadth. As with the GORG database, less than 1% of these genomes were both producers and consumers (*SI Appendix*, Fig. S2). All genomes with both traits had high-scoring hits to phosphonate production and consumption genes. Although we identified the SAR11 strain HTCC7217 and two marine Roseobacter isolates (*Actibacterium atlanticum* 22II-S11-z10 and *Pseudooceanicola nanhaiensis* DSM 18065) with both a C-P lyase gene cluster and phosphonate biosynthesis genes, the vast majority of genomes with both production and consumption traits in this dataset were nonmarine (Dataset S3). For example, almost half were from the Burkholderiaceae family of the Betaproteobacteria (*SI Appendix*, Fig. S2), which included many human pathogens. Thus, the general mutual exclusivity between phosphonate biosynthesis and catabolism traits is not unique to the marine environment. In surface ocean clades of *Prochlorococcus* and SAR11, this mutual exclusivity occurs at the species/strain level, which implies strong functional differentiation between closely related cells (Fig. 2 *C* and *D*) and fine-scale niche partitioning with regard to phosphonate function and may reflect functional incompatibility or ecological/evolutionary tradeoffs between biosynthesis and catabolism.

### Phosphonate Production Traits Are Horizontally Transferred.

Because phosphonate production and consumption traits are found in a subset of the genomes from each taxonomic group (*SI Appendix*, Table S1), they are part of their flexible genome—as has already been demonstrated for both SAR11 and *Prochlorocccus* (28). Horizontal gene exchange is a key mechanism maintaining genes at medium-low frequencies within the flexible genome of microbial populations (29); thus, we looked for its signatures with regard to phosphonate production genes. We found that their gene-flow patterns do not follow a tree-like pattern and are better explained by horizontal exchange and gene loss. The topological distance between a species phylogeny and a *pepM* phylogeny approaches values for random tree sets and is over twice the distance between the species tree and trees from conserved metabolic gene families (*SI Appendix*, Fig. S3). Thus, there is a poor correlation between species trees, inferred from conserved marker genes, and trees constructed from the *pepM* gene—a hallmark of horizontal gene transfer (30). Furthermore, nearly all phosphonate biosynthesis gene cassettes are located within “genomic islands'' in *Prochlorococcus* (*SI Appendix*, Fig. S4), which contain the majority of laterally transferred genes in this genus (31, 32). There is also a transposase—an important molecular mechanism of horizontal gene transfer in bacteria (33)—four genes downstream of *pepM* in the *Prochlorococcus* SB genome (*SI Appendix*, Fig. S5). Overall, these findings suggest that the phosphonate production trait has been frequently exchanged among marine microbial communities.

### Oceanographic Trends in Phosphonate-Producing Microbes.

**Fig. 3.**
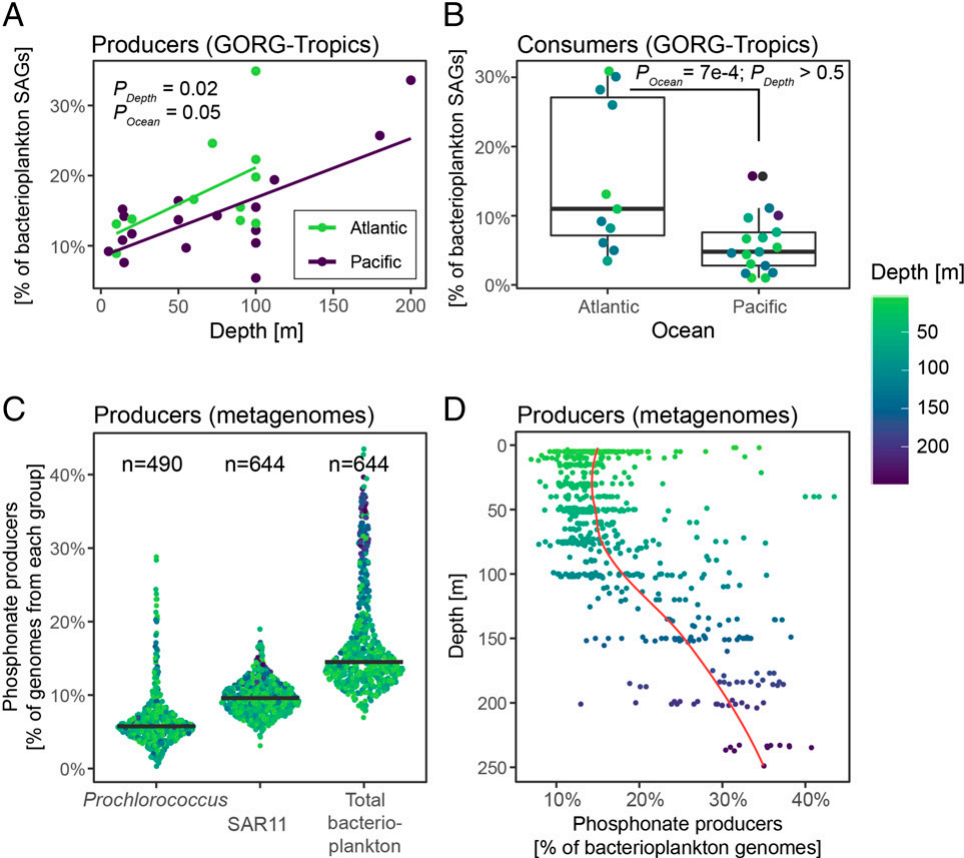
Oceanographic drivers of phosphonate production. (*A*) The proportion of phosphonate producers significantly increases with depth in the 28 GORG-Tropics samples (beta-binomial regression; Est = 0.004, Err = 0.001, t = 2.604, and *P* = 0.02; link = logit; log L = −78.179, df = 4, and resid df = 24). Producers are slightly more common in the Atlantic than the Pacific (beta-binomial regression; Est = −0.30, Err = 0.14, t = −2.08, and *P* = 0.05; link = logit; log L = −78.179, df = 4, and resid df = 24). Each point is a sample (median SAGs per sample = 241). (*B*) Phosphonate consumers in the Atlantic and Pacific oceans as percent of all SAGs (Est = −1.04, Err = 0.27, t = −3.81, and *P* = 7 × 10^−4^; link = logit; log L = −80.425, df = 4, and resid df = 24). Proportions (%) are total producers or consumers divided by the total number GORG assemblies and are corrected using the estimated sequence recovery from assemblies (*Materials and Methods*). (*C*) Percentage of phosphonate producers in the global (sub)tropical surface ocean estimated from BioGEOTRACES and Tara Oceans metagenomes. Total bacterioplankton category includes both Bacteria and Archaea. Black line is the median value for n metagenome samples for each taxonomic group. (*D*) The relationship between depth and percent of all bacterioplankton with the phosphonate production trait. The red line is a simple Loess regression fit to the data.

How do the global distributions of putative phosphonate producers and consumers relate to hydrographic and biogeochemical patterns? Results from GORG-Tropics revealed two significant trends: 1) the abundance of putative phosphonate producers significantly increases with depth and is slightly higher in the Atlantic Ocean (Fig. 3*A* and *SI Appendix*, Fig. S6) and 2) putative phosphonate consumers, including both SAR11 and *Prochlorococcus*, are significantly more abundant in the Atlantic than in the Pacific Ocean (Fig. 3*B* and *SI Appendix*, Fig. S6), which is consistent with past reports (27, 34). We found very high consumer abundance in four samples from the northwestern edge of the Sargasso Sea that were collected during late autumn. However, even after excluding these four samples the Pacific/Atlantic difference was still significant (*P* = 0.02). We then greatly increased the temporal and spatial resolution of our analysis by using nearly 700 metagenomes from GEOTRACES, Tara Oceans, and two long-term ocean time series sites (35–37) in order to look for other relationships between putative phosphonate producers and environmental variables. Based on metagenome read recruitment to *pepM* and conserved marker genes (*Materials and Methods*), we estimate that 15% of bacterioplankton genomes are putative phosphonate producers (Fig. 3*C*). A total 6% of *Prochlorococcus* carry the trait, as do 10% of SAR11 (Fig. 3*C*), corroborating the numbers we found in the GORG-Tropics SAGs (*SI Appendix*, Table S1). Likewise, the dominant trends we observed in GORG-Tropics were clearly recovered by the metagenomes. There was no statistically significant difference in producer abundance between ocean basins (*SI Appendix*, Fig. S7*A*), and the abundance of cells carrying phosphonate production genes increased with depth, with a rapid increase starting near 100 m (Fig. 3*D* and *SI Appendix*, Fig. S8). The agreement between the metagenome and single-cell genome–resolved approaches highlights the robustness of our findings.

To better understand what drives the distribution of phosphonate producers in the sunlit ocean, we next incorporated these metagenomic datasets along with chemical, biological, and hydrographic data into predictive models. The models suggest that on average, *Prochlorococcus* phosphonate producers are most abundant where inorganic phosphate and DOP concentrations are highest (*SI Appendix*, Figs. S7 and S8). For example, the North Atlantic and Mediterranean had the lowest median abundance of *Prochlorococcus* producers. In GORG-BATS (Bermuda Atlantic Time-series Study), we detected no *Prochlorococcus* phosphonate producer genomes even though the sample was deeply sequenced (>6,000 genomes). We interpret this as reflecting potential selection against the phosphonate biosynthesis trait in *Prochlorococcus* populations from consistently P-limited regions due to the additional P cost of phosphonate biosynthesis. The relative abundances of SAR11 producers and total producers were shaped by many of the same factors consistent with SAR11 being the dominant phosphonate producer in GORG-tropics and GORG-BATS. We found that the greatest amount of phosphonate producer variation is explained by multiple depth-dependent biotic factors (*SI Appendix*, Figs. S7 and S8). Overall, the models predict that phosphonate producers are most abundant in the lower reaches of the euphotic zone and upper reaches of the mesopelagic. Future chemical studies targeting the 100- to 300-m depth range will hopefully provide greater understanding of phosphonate production in the upper ocean.

We next looked for temporal patterns from metagenomes collected from the Hawaii Ocean Time-series (HOT) and the BATS, two long-running time series representative of the North Pacific and North Atlantic subtropical gyres (38, 39). BATS has a lower-total dissolved P inventory than HOT, which has been linked to variation in P-related gene content in BATS *Prochlorococcus* populations (28). Putative phosphonate producer abundance peaked during late winter and early spring at BATS, coinciding with wind-driven deep mixing (*SI Appendix*, Fig. S9*C*), which increases the supply of inorganic phosphate to the surface waters (40) and may also recruit phosphonate producers from deeper layers. Similarly, phosphonate-producing SAR11 ecotypes IIa and IIb are abundant in the winter/spring (41), while phosphonate-consuming SAR11 ecotypes IV and V dominate in the highly stratified summer months when inorganic phosphate concentrations are low (Fig. 2*D*). Likewise, putative *Prochlorococcus* producers declined to negligible levels in the summer. This implies that the phosphonate production trait occurs at the highest frequency when inorganic phosphate concentrations are relatively high, possibly reflecting a high cost of production.

Based on the seasonal results at BATS and our intuition that phosphonate production might increase overall cellular P demand, we asked whether the phosphonate production trait was less abundant in low P regions of the ocean or whether the trait was associated with other oceanographic features. We found a small but significant positive relationship between inorganic phosphate and DOP concentrations and the abundance of *Prochlorococcus* phosphonate producers (*SI Appendix*, Figs. S7*B* and S9 and Table S2 and *SI Results*). This is consistent with the seasonal variation of phosphonate producers at BATS and implies that relatively high inorganic phosphate and DOP conditions support more cells with genes encoding for phosphonate production. However, we did not observe a relationship between inorganic phosphate or DOP and putative SAR11 or total community producers. Thus, the relationship between community-averaged phosphonate production and oceanographic P inventories is unclear. For *Prochlorococcus*, at least, higher-total dissolved P concentrations seem to be associated with a higher fraction of cells with the genetic potential for phosphonate production.

Exploring the depth dependence of phosphonate producers yielded a few additional insights. In samples shallower than 100 m, a median of 15% of bacterioplankton have phosphonate biosynthesis genes. That proportion steadily increases with depth to nearly 30 to 40% at 200 m (Fig. 3D). The 100-m inflection coincides with the nutrient-driven “genomic transition zone” (i.e., a region where bacteria and archaea tend to have larger genomes with higher GC content and proteins with higher N/C ratios) (42). Again, nutrient availability, which increases with depth, is an important factor in the relative abundance of putative phosphonate producers. Finally, the proportion of putative phosphonate producers in the upper 100 m of the tropical ocean is relatively modest (<15%). Since phosphonates appear to be relatively rapidly cycled (18) and constitute a significant fraction of the DOP, this implies that cellular production rates would need to be relatively high to account for the large global inventory of phosphonates in marine dissolved organic matter.

### Phosphonate Production in *Prochlorococcus* SB.

**Fig. 4.**
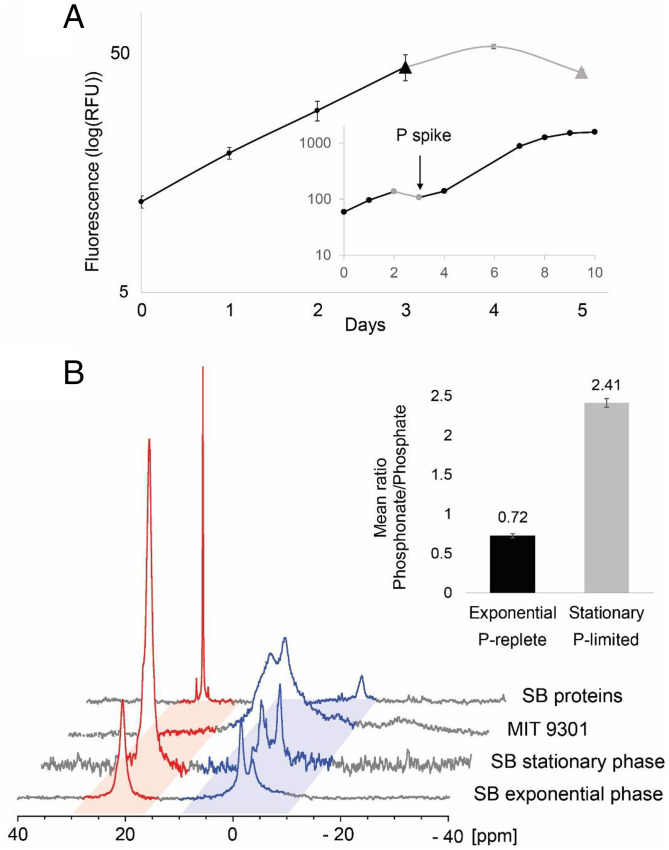
Phosphonate production by cultured *Prochlorococcus* SB. (*A*) *Prochlorococcus* SB growth curve in medium with N/*P* = 350/1 showing exponential phase (P-replete; black line and symbols) and stationary phase (P-starved; gray line and symbols) due to P-starvation. Triangle data points correspond to the days the cells were harvested in the different growth phases. Error bars are calculated based on the SD between the biological duplicates. The *Inset* represents the *Prochlorococcus* SB growth curve in medium with N/*P* = 350/1 in which inorganic phosphate was added on Day 3 to reach N/*P* = 16/1. (*B*) ^31^P-NMR spectra of exponential (P-replete) and stationary (P-starved) phase *Prochlorococcus* SB whole cells, P-replete Prochlorococcus MIT9301 whole cells, and the insoluble protein fraction of P-replete *Prochlorococcus* SB. The phosphonate and phosphate regions of the spectra are indicated in red and blue, respectively. While *Prochlorococcus* SB produces phosphonate and doubles its relative phosphonate content in P-starved stationary phase, the negative control, MIT9301 only produces phosphates. The histogram (*Inset*) displays the mean phosphonate/phosphate ratios for *Prochlorococcus* SB cells harvested in exponential (black) and stationary (gray) phase calculated by integrating the phosphonate and phosphate peaks in *Prochlorococcus* SB whole-cell ^31^P-NMR spectra obtained for the duplicates in each growth phase. Error bars correspond to the SD of the biological replicate.

To better understand how much cellular P is allocated to phosphonates and with which macromolecules they are associated, we sought to examine phosphonate production by an abundant, widespread, and experimentally tractable marine microbe. Only one cultured isolate meets these criteria: *Prochlorococcus* SB. Since there is no genetic system for knockout mutants in *Prochlorococcus*, we used *Prochlorococcus* MIT9301, a closely related strain lacking the phosphonate biosynthesis cluster, for comparison. Using ^31^P NMR spectroscopy (^31^P-NMR), we found that under P-replete conditions *Prochlorococcus* MIT9301 allocates all its P to phosphate and pyrophosphate esters (−12 to 12 ppm; Fig. 4*B*). In contrast, *Prochlorococcus* SB—the strain with the phosphonate biosynthetic pathway—produces both phosphate and pyrophosphate esters as well as phosphonate esters (43) (18 to 27 ppm; Fig. 4*B*). Some cyclic phosphates, specifically 1, 3, 2-dioxaphospholanes such as 2’, 3′-cyclic nucleotide monophosphates, have chemical shifts in the 14- to 20-ppm spectral region and could be misassigned as phosphonates based on their ^31^P-NMR spectra alone. We therefore treated *Prochlorococcus* SB with 0.1 N NaOH using conditions that are known to degrade 2’,3′-cyclic nucleotides. ^31^P-NMR spectra collected before and after base-catalyzed hydrolysis showed no measurable difference in the integrals of the phosphonate/phosphate ester regions, indicating that 2’, 3′-cyclic nucleotide monophosphates and similar 1, 3, 2-dioxaphospholanes do not contribute significantly to P signal in the 18- to 27-ppm region of the ^31^P-NMR spectra (*SI Appendix*, *SI Results* and Fig. S12). Integration of the phosphate and phosphonate ester regions of the NMR spectrum indicated that under nutrient replete conditions, *Prochlorococcus* SB cells allocate ∼40% of their cellular P to phosphonate production (Fig. 4*B* and *SI Appendix*, Table S3).

### Phosphonates and P Storage.

The physiological and ecological roles of phosphonates are poorly understood. However, the presence of *pepM* among diverse, genome-streamlined bacteria and the high relative abundance of cellular phosphonates in *Prochlorococcus* SB suggest that phosphonates serve an essential function for microbes inhabiting oligotrophic marine waters. In highly stratified oligotrophic waters, nutrient supply to phytoplankton is episodic, driven by mixing events that bring nutrient-rich waters from below the surface into the euphotic zone. Some microbes take up excess P during periods of high nutrient concentrations and sequester them for later use (5, 6); thus, one function that has been suggested for phosphonates is that they could serve as an intracellular P-storage reservoir (44). The *Prochlorococcus* SB genome, however, lacks phosphonate degradation pathways, including C-P lyase, phosphonate hydrolytic pathways (10), and PhnYZ, an oxidative pathway recently discovered in another strain of *Prochlorococcus* (27).

Recognizing that *Prochlorococcus* SB might use an uncharacterized pathway to repurpose P from phosphonates, we tested the P-storage hypothesis in this strain by comparing phosphonate production in cultures grown under P-starved and P-replete conditions (Fig. 4*A*), expecting that if phosphonates were used for P storage, P starvation would reduce the allocation of P to phosphonates. We found, however, that this strain allocates significantly more P to phosphonates relative to phosphates upon entering P-starved stationary phase (phosphonate/phosphate = 2.4) than during P-replete exponential growth (phosphonate/phosphate = 0.72; Fig. 4*B*). Cellular C-P was relatively stable across exponential and stationary phases (145 and 131, respectively); changes in the phosphonate/phosphate ratio were driven by a decrease in phosphate ester content during P starvation, while the cells kept producing phosphonates (*SI Appendix*, Fig. S10 and Table S4). Thus, P allocated to phosphonates is not internally recycled to sustain growth during periods of P starvation, consistent with our observation that *Prochlorococcus* SB lacks any known pathway to degrade and assimilate phosphonates.

### Macromolecular Forms of Phosphonates.

The biochemical function of phosphonates is likely linked to their macromolecular form; thus, we explored this in *Prochlorococcus* SB cultures. We expected phosphonates to be associated with polysaccharide macromolecules, because phosphonate biosynthesis genes in many *Prochlorococcus* and SAR11 genomes are surrounded with glycosylating enzymes predicted to be involved in the biosynthesis of large extracellular polysaccharide structures, such as bacterial capsules (*SI Appendix*, Fig. S5 and Table S5). Further, phosphonates are constituents of the large reservoir of dissolved polysaccharides that accumulate in the surface ocean as HMWDOP (18). However, after fractionating *Prochlorococcus* SB cells into major biochemical classes (*Materials and Methods*), we found that phosphonates were recovered not in the polysaccharide fraction but rather in the protein fraction (Fig. 4*B*)—more specifically within the methanol/acetone insoluble HMW protein fraction, which includes membrane-associated proteins. We reconcile this mismatch between expectation and findings by proposing that phosphonates are integrated into glycan polymer chains that are then posttranslationally attached to HMW membrane-anchored proteins (*SI Appendix*, Fig. S5 *A*–*D* and Table S5 and *SI Results*).

One of the most-common posttranslational modifications of bacterial proteins is O-linked glycosylation (45–48), in which glycan polymers are covalently bound to larger protein complexes. The phosphonate gene clusters in *Prochlorococcus* SB and two SAR11 isolate genomes are all within ± 5 Kbp of both glycan assembly enzymes and lipid carriers predicted to be involved in the biosynthesis of polysaccharide capsules (*SI Appendix*, Fig. S5 *B*–*D* and Table S5). In some bacteria, the glycan building blocks for capsule biosynthesis are also routed toward posttranslational O-linked glycosylation of membrane-bound lipoproteins (49). The *Prochlorococcus* SB phosphonate gene cluster contains an initiating glycotransferase (PgIC) that links the first glycan subunit to a lipid carrier, a flippase (Wzx) that translocates the assembled glycan-bound lipid carrier to the periplasmic face, and an O-oligosaccharyltransferase (PgIL) that is the critical enzyme moving the assembled glycan chain to the final acceptor (48, 49). The PgIL family has recently been demonstrated to transfer preassembled glycan chains onto protein substrates (50, 51). These O-oligosaccharyltransferase genes are also present near *pepM* in SAR11 genomes (*SI Appendix*, Fig. S5), and 15% of GORG-Tropics SAGs contain *pepM* plus an O-oligosaccharyltransferase-like domain colocalized within the same 10-Kbp genome segment. This proportion increases to 50% if we relax the condition that the genes must occur on the same genomic contig. Thus, genomic evidence implies that phosphonates could be a common moiety involved in the posttranslational O-linked glycosylation of proteins in the ocean.

### Functional Roles of Phosphonylated Glycoproteins.

The widespread distribution of phosphonate synthesis genes, both taxonomically and geographically, the abundance of phosphonates in HMWDOP, and the high relative allocation of P into phosphonates in *Prochlorococcus* SB suggest that phosphonate production confers an important fitness benefit. As we argue above (*Phosphonates and P Storage*), it would appear that this benefit is not related to P nutrient storage. Rather, we hypothesize, based on several lines of inference, that this fitness advantage is linked to cell-surface defenses that mitigate mortality caused by viral lysis or protozoan grazing—two significant drivers of mortality for *Prochlorococcus* (52).

First, as detailed previously, the phosphonate biosynthesis genes are frequently flanked by genes for the biosynthesis of extracellular structures such as membrane-anchored polysaccharides. This genomic organization suggests that phosphonates are linked to polysaccharides that are posttranslationally attached to protein at the cell surface, interacting with the external environment. Many marine microbes produce extracellular layers of polysaccharides to defend against predation (53) and surface-expressed structures protect against enzymatic attack, ultraviolet radiation, phages (54–56), and protozoan grazers (57–60). Specifically, cell-surface structure modification is a common strategy to prevent phage attachment, block the injection of phage DNA (61), or influence the biochemistry of those structures. Modifications can change membrane hydrophobicity, which has been shown to influence the ingestion of *Prochlorococcus* by a nanoflagellate grazer (62), and provoke active rejection of prey or prevent digestion by inhibiting some enzymes (63). Phosphonates are often incorporated into cell-surface structures, because they protect the cell exterior from hydrolysis (64). For example, phosphonates have been found in polysaccharide B (65–68) or as a glycoprotein modification in higher organisms (69, 70). Although the bioactivity of methylphosphonate and 2-hydroxyethylphosphonate, the most-abundant phosphonates in HMWDOP, has not been investigated, many phosphonates are potent antibiotics and highly bioactive. Indeed, they can mimic phosphate and carboxylic acid functional groups, thereby interrupting important metabolic pathways (10, 71).

Second, the phosphonate biosynthesis trait in *Prochlorococcus* and SAR11 populations is present at low frequency in the wild, and its distribution is consistent with the evolutionary mechanism of negative frequency-dependent selection, which favors the presence of rare traits in a population (29). Negative frequency-dependent patterns are well documented for microbial cell-surface structures, including lipopolysaccharides, S-layers, O-antigens, and capsular polysaccharides (72). Rare variations in these surface structure traits—for example, slight structural modification—can facilitate bacterial evasion from phage and host immune responses (73, 74). However, once a variant becomes too abundant within a population, then predators, phages, or the immune system may adapt to the variant, neutralizing any conferred advantage. Thus, natural selection in these defense traits should result in rapid gene gain and loss, which ultimately makes these genes and their variants uncommon in the wider population.

**Fig. 5.**
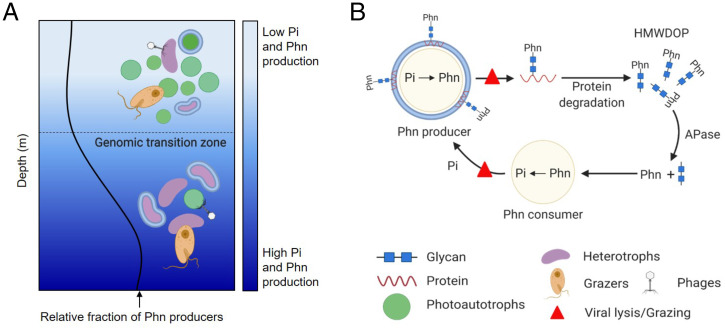
Hypothesized microbial cycling of phosphonates in the surface ocean. (*A*) In the surface ocean, phosphate (Pi) is scarce and often limiting. Producing phosphonate (Phn) as a mortality defense may be costly in terms of resource allocation. Therefore, the relative fraction of phosphonate producers in the microbial community is low. As nutrient availability increases through the genomic transition zone (42), the P cost to produce phosphonate decreases and the benefit/cost ratio of phosphonate production, in which the benefit is a decrease in mortality, increases causing phosphonate producers to be relatively more abundant. (*B*) Microbes with PepM produce cell surface-layer phosphonoglycoproteins to reduce mortality from grazing or viral infection. Upon death of the cell, phosphonoglycoproteins are released into seawater where heterotrophic microbes quickly remineralize proteins leaving phosphonoglycans to accumulate as HMWDOP. Phosphonates are hydrolyzed from glycans by alkaline phosphatase (APase) family enzymes and further hydrolyzed into phosphate by C-P lyase or other hydrolytic pathways. Recycled phosphate can then be used to produce new phosphonates.

The depth distribution of phosphonate biosynthesis trait is consistent with the hypothesis that phosphonate might be used as a defense mechanism, as the relative abundance of phosphonate producers is relatively low in the surface and increases with depth, peaking at the nutrient-driven genomic transition zone/deep chlorophyll maximum layer (DCM). We hypothesize that chronic, long-term P scarcity drives the cost of phosphonate synthesis to outweigh its fitness benefit for most bacterioplankton in oligotrophic surface communities, resulting in low frequency of the phosphonate biosynthesis trait among community members. In contrast, at depths near the genomic transition zone/DCM, the greater availability of inorganic phosphate may lower the relative cost of phosphonate production while higher densities and diversity of phages (75, 76), and nanoflagellate grazers (77–79)—key grazers of *Prochlorococcus* (80) and heterotrophic bacteria (81–83)—may simultaneously increase the relative benefit of cell-surface modification with phosphonates (Fig. 5*A*).

### Rare Traits Influence Major Biogeochemical Cycles.

What are the consequences of phosphonate production on nutrient and other biogeochemical cycles? The depth distribution of the phosphonate synthesis trait (i.e., low abundance in the surface and higher at the DCM) mirrors P-reduction rates in the euphotic zone (84), which implies that phosphonate production is correlated to the abundance of phosphonate synthesis marker genes. This explains the virtual lack of phosphonates in surface particulate matter samples (84, 85) and implies that the amount of phosphonate in particulate organic matter should be higher at the DCM. At steady state, low rates of phosphonate production would be balanced by low rates of phosphonate degradation (C-P lyase activity) (86) and export (87). Similar reasoning argues that higher phosphonate production rates around the DCM would sustain higher overall rates of phosphonate degradation, consistent with the elevated C-P lyase gene copy numbers and C-P lyase activity measured in and below the DCM (27, 86). This implies that P redox-cycling is more active at depth than in the surface ocean. Under this scenario, open-ocean methane production by the C-P lyase degradation pathway is directly coupled to the fitness benefit of phosphonate production. This should lead to increased production of methylphosphonate, and ultimately methane, with depth, which is consistent with observations in the North Atlantic and North Pacific (88, 89). Across large areas of the ocean, this depth dependence of methylphosphonate production would in turn influence the fraction of total methane that either escapes to the atmosphere or is oxidized by methane-oxidizing bacteria (90).

If, as we hypothesize, phosphonates are used to modify cell-surface glycoproteins, the cycling of these glycoproteins might explain why phosphonates are mainly observed in HMW dissolved organic matter (i.e., linked to polysaccharides). We propose that after cell death, the labile protein component of the phosphonylated glycoproteins likely turns over quickly. However, inherently slow degradation of the structural polysaccharide component would lead to the accumulation of phosphonoglycans as HMW dissolved organic matter (91), explaining their abundance in marine DOP (Fig. 5*B*). Unlike for the majority of DOP that emerges from primary metabolism (e.g., nucleic acids, phospholipids, and energy currency) the relatively high concentrations of phosphonoglycans is a consequence of ecological interactions such as the defense mechanism we propose here.

MGI Thaumarchaeota (21), *Trichodesmium* (20), and *Prochlorococcus* (this study) isolates have all been shown to produce phosphonates. However, these groups occur at very different densities in the sunlit oligotrophic ocean. MGI Thaumarchaeota are generally low abundance (∼5 × 10^4^ cells/L^−1^) (92–94) but rapidly increase with depth in the upper mesopelagic (95). The raft morphology of *Trichodesmium* can occur at 60 colonies/m^−3^ in nonbloom conditions (96), while mean *Prochlorococcus* abundance is 4.4 × 10^7^ cells/L^−1^ between 30°N and 30°S (97). Extrapolating from these cell densities, the fraction of each group with phosphonate production potential (this study), published P quotas from culture and field experiments (98–100), and average phosphonate content (20, 21), we estimate that MGI Thaumarchaeota, *Trichodesmium*, and *Prochlorococcus* could respectively account for <1%, <20%, and >60% of particulate phosphonate production in the global euphotic zone (*SI Appendix*, *SI Results*). Even though phosphonate production has not been experimentally confirmed in SAR11, it is plausible that SAR11 isolates have a cellular phosphonate content similar to *Trichodesmium* or *Prochlorococus*. Assuming SAR11 phosphonate content is 10% of total cellular P (similar to *Trichodesmium*) and that 18% or 9.9 × 10^26^ cells of SAR11 in the sunlit ocean are phosphonate producers (25, 101) with P quotas of 0.16 × 10^−16^ mol P/cell^−1^ (102), we estimate that SAR11 could easily account for 75% of the phosphonate budget in the sunlit ocean (*SI Appendix*, *SI Results*). Phosphonates, produced by only a minor proportion of all marine microbes, constitute a relatively large bioavailable P pool in the upper ocean. Thus, rare biosynthetic traits in numerically abundant microbes such as *Prochlorococcus* and SAR11 can have significant ecological implications for global biogeochemical cycles.

## Materials and Methods

Refer to *SI Appendix* for detailed methods descriptions.

### Genomic Data Sources.

We used two collections of genomes for the comparative genomics analysis: MARMICRODB (103) (https://zenodo.org/record/3520509) and the GORG-Tropics dataset (24). We also used 195 surface and DCM metagenomes from Tara Oceans project (36) and 480 metagenomes from bioGEOTRACES, HOT, and BATS (35). Genome and metagenome quality control and exclusion criteria were performed as described earlier (103).

### Homology Searches.

Phosphonate biosynthesis and catabolism proteins were identified by homology to a collection of Hidden Markov Models using HMMER version 3.1b2 (http://hmmer.org/) and the trusted cutoffs of each individual model. We followed an earlier approach (22) with some modifications. Briefly, we considered any genome with a valid *pepM* sequence (determined by the presence of the catalytic motif “EDK(X)5NS”) as a potential phosphonate producer. We did not require the presence of *ppd*, *pdh*, or *mpnS* genes for a complete phosphonate biosynthetic pathway; we only recorded the presence of these genes if they were adjacent to *pepM*. Most *pepM*-containing genomes did have at least the decarboxylase adjacent to the *pepM* sequence or on a different contig if the cluster was on a contig break. For phosphonate catabolism proteins, gene neighborhoods were inspected to confirm the presence of multiple co-occurring genes from the PhnYZ (15), C-P lyase, 2-aminoethylphosphonate, and phosphonoacetaldehyde phosphonatase catabolism pathways. We also identified two sets of 10 highly conserved, single-copy families from *Prochlorococcus* and SAR11 lineages for use in normalization with metagenome profiling. The protein sequence profiles, results from these searches, and descriptions of the sequence families are available from https://doi.org/10.5281/zenodo.5117752. Full details are in *SI Appendix*, *Detailed Methods*.

### Multiple Sequence Alignments, Phylogenetic Inference, and Topological Comparison.

We aligned authentic PepM peptide sequences containing the “EDK (X)5NS” motif to a PepM hidden Markov model (TIGR02320), and we manually inspected the alignments to ensure the veracity of the “EDK (X)5NS” motif. We created the multi-phylum genome phylogenies from genome assemblies with confirmed phosphonate biosynthesis pathway or at least one confirmed phosphonate catabolic pathway. We used the Genome Taxonomy Database (GTDB) Toolkit version 1.3.0 pipeline (104) with default settings and the GTDB R05-RS95 database (105) to identify conserved proteins (120 bacterial proteins/122 archaeal proteins) and generate concatenated multiprotein alignments. The multi-phylum genome phylogenies and the PepM phylogeny were inferred using FastTree version 2.1.10 (106). Detailed phylogenies of *Prochlorococcus* and *Pelagibacterales*/SAR11 in *SI Appendix* were constructed as described earlier (103). We used the Mutual Clustering Information, a generalized Robinson–Foulds metric (107), for comparing the topologies of the PepM phylogeny, the genome phylogeny, and conserved gene family trees as implemented in the TreeDist version 2.0.3 R package.

### Gene Enrichment Analysis.

We annotated the 16 *Prochlorococcus* and 22 SAR11/*Pelagibacterales* genomes containing verified *pepM* sequences with eggNOG 4.5.1 (108) using eggNOG-Mapper version 1.0.3–3-g3e22728 (109). Significant enrichment of Kyoto Encyclopedia of Genes and Genomes pathways was determined using the hypergeometric test (110) implemented in clusterProfiler version 3.8 (111).

### Identification of *pepM* Sequences within *Prochlorococcus* Genomic Islands.

We predicted genomic islands using Hidden Markov Models trained from conserved gene synteny patterns in closed *Prochlorococcus* genomes as done earlier (112).

### Estimation of *pepM* Prevalence in *Prochlorococcus* and SAR11 Genomes.

We attempted to estimate the “true” proportion of genomes with *pepM* while correcting for genome incompleteness. Briefly, we used the estimated genome completeness from checkM version 1.0.11 (113) to estimate the number of missing bases per taxonomic group and then used this to scale the relative abundance of potential phosphonate producers per group. We estimated the corrected prevalence of phosphonate producers as:

$$\left(\frac{\sum_{i=1}^{n}p_{i}}{\sum_{i=1}^{n}g_{i}}\right)\;\times \sum_{i=1}^{n}\left(\frac{g_{i}}{c_{i}}\right)\times {\bar{p}}^{-1}\times n^{-1},$$

where for each clade, *p* is the length of the phosphonate biosynthesis/degradation operon in base pairs, *g* is the length of the genome assembly in base pairs, *c* is the completeness estimate from CheckM, *p* is the average length of all phosphonate biosynthesis/degradation operons from each clade, and *n* is the total number of assemblies from each clade. For the GORG datasets, we applied no additional completeness cutoffs over what was used in the original publication. Applying the commonly used completeness cutoff of 30% did not change the outcome.

### Metagenome Read Classification.

We identified metagenome reads derived from marker genes as reciprocal best hits using Diamond version 0.9.22.123 (114). We identified reads derived from *pepM* using empirically determined score cutoffs for the *pepM* genes present in MARMICRODB. To normalize *pepM* reads across metagenomes, we used the length normalized abundance of the 10 single-copy marker genes from *Prochlorococcus*, SAR11, and all bacteria and archaea in each metagenome.

### Biotic and Abiotic Data Associated with Metagenomes.

The biotic and abiotic variables used in this study were obtained and preprocessed as described in detail here: https://doi.org/10.5281/zenodo.3689249 and here: https://doi.org/10.5281/zenodo.3786232. We obtained inorganic phosphate concentrations from the GEOTRACES Intermediate Data Product IDP2017 version 3 (37), specifically from sections GA02 (115, 116), GA03, GA10 (117), and GP13. We obtained inorganic phosphate concentrations from the Tara Oceans project (118) (https://doi.pangaea.de/10.1594/PANGAEA.875579). Modeled climatological DOP and other variables were obtained from the Massachusetts Institute of Technology Darwin model (version 0.1_llc90; darwinproject.mit.edu/) and from the Simons Collaborative Marine Atlas project (119). Taxonomic profiles derived from metagenomic reads were generated as described earlier (103) using the MARMICRODB database.

### Data Analysis.

We fit one random forest model for *pepM* relative abundance from *Prochlorococcus*, SAR11, and bacteria plus archaea. Each model was trained using up to 44 abiotic/biotic variables including trace metal and macronutrient data from GEOTRACES and Tara Oceans, modeled climatological means from the MIT Darwin model (https://darwinproject.mit.edu/), and ecotype relative abundances. Random forest regression was implemented with the R package Ranger (120). We determined predictor variable rankings on the final model from the cross-validation step using the Boruta heuristic (121). This step allowed us to identify all predictor variables that consistently performed better than chance and to compare the importance of each variable to a reference importance level (i.e., random data). We used the R package corncob for beta-binomial regression (122) and modeled *pepM* relative abundance directly from *pepM* read counts and the median read counts to marker gene. We estimated seasonal effects in time series metagenomes using Generalized Additive Mixed Models and Linear Mixed-Effect Models implemented through the mgcv version 1.8–26 (84) nlme version 3.1–148 libraries in R v3.6.2.

### *Prochlorococcus* Cultures under P-Replete and P-Starvation Conditions.

We axenically grew *Prochlorococcus* SB and *Prochlorococcus* MIT9301 under constant light (30 μmol/quanta/m^−2^/s^−1^) in artificial seawater medium AMP1 prepared has described earlier (123) but using 3.75 μM [tris(hydroxymethyl)methylamino]propanesulfonic acid (TAPS) as a buffer instead of 1 mM Hepes and a phosphate concentration of 50 µM (N/*P* = 16/1) and 2.28 µM (N/*P* = 350/1) to study the effect of P starvation. We documented the P-starvation state by adding phosphate to the culture and showing that growth resumed (Fig. 4*A*). We assessed the culture’s axenicity by flow cytometry and by confirming a lack of turbidity for at least 30 d after inoculation with three purity broths: ProAC (124), MPTB (125), and ProMM (126). We cleaned all the glassware and polycarbonate bottles by soaking overnight in 2% detergent, rinsed six times with deionized water, soaked overnight in 1 M HCl, and rinsed six times with ultra–high purity water.

### Cell Harvest and Treatment.

We harvested the *Prochlorococcus* SB cultures grown in high N/P medium twice: ∼6 L during exponential growth and the remainder (14 L) harvested 2 d after the onset of stationary phase growth. We separated the cells from the growth medium by centrifugation (15,970 rcf for 30 min at 4 °C). After rinsing, we flash-froze the cells in liquid nitrogen and stored them at −20 °C until we conducted the NMR analyses.

### NMR.

We acquired the NMR spectra of whole cells at 25 °C on a 400-MHz Bruker AVANCE DPX spectrometer using a 5-mm inverse broadband probe and running TopSpin 1.3. ^31^P shifts are reported relative to external 85% phosphoric acid at 0 ppm. For the proton-decoupled ^31^P-NMR spectra, we used “zgdc30” with WALTZ16 decoupling and sweep width of 80 ppm, a 3-s relaxation delay, 100K scans, and 20-Hz line-broadening. We packed the *Prochlorococcus* SB and MIT9301 whole cells into a 5-mm tube (Shigemi Inc.) with magnetic susceptibility of the glass inserts matching D_2_O. We acquired the ^31^P-NMR spectra of *Prochlorococcus* SB protein fraction at 25 °C on a 400-MHz Bruker Ascend 400 equipped with a Sample CASE and using the program “zgpg30” with a sweep width of 80 ppm, a relaxation delay of 2 s, a 15-Hz line-broadening, and for 13K scans. ^31^P chemical shifts are reported relative to external phosphoric acid at 0 ppm.

### Elemental Composition of *Prochlorococcus* SB and MIT9301.

Elemental C/N/P ratios were measured at the University of Hawai'i nutrient facility according to the protocols employed by the HOT program (https://hahana.soest.hawaii.edu/hot/methods/pcpn.html). Cell pellets from ∼900 mL of culture were transferred to combusted glass vials, dried, and powdered. C and N were measured on subsamples using a PE-2400 Carbon/Nitrogen analyzer calibrated with acetanilide standards. Cellular P was measured by the molybdenum blue method (127, 128) after combusting cell pellets at 450 °C for 3 h and dissolving the residue in 10 mL of 0.5 M HCl.

### Separation of Cellular, Macromolecular Classes.

To fractionate *Prochlorococcus* SB organic matter into different classes of major biochemicals, we followed the protocols of Sosa et al., 2019 (15). Cells from 3 L of culture (∼2.5 × 10^11^ cells/L) were centrifuged (12,100 rcf; 20 min) at 4 °C. The supernatant was carefully decanted, the cell pellets and concentrates combined and transferred to a 15-mL centrifuge tube, and the final suspension pelleted (12,100 rcf; 20 min) at 4 °C. The supernatant was carefully removed by pipette and the cell pellet suspended in 5 mL of cold (4 °C) 5% aqueous trichloroacetic acid (aqTCA). The suspension was agitated for 1 h, after which the mixture was centrifuged (12,100 rcf; 30 min at 4 °C) and the supernatant recovered by pipette. The aqTCA insoluble material was extracted two additional times by suspension in 5% cold (4 °C) aqTCA followed by centrifugation (12,100 rcf; 30 min at 4 °C). The aqTCA fractions were combined and evaporated to dryness by rotary evaporation to recover the polysaccharide (carbohydrate) fraction (*SI Appendix*, Fig. S13). TCA-insoluble material was suspended in 5 mL 95% aqueous ethanol (aqEtOH), manually agitated at room temperature for 20 min, and centrifuged (12,100 rcf, 30 min) and the supernatant recovered by pipette. The aqEtOH extraction was repeated two additional times, centrifuged each time (12,100 rcf; 30 min), and the supernatants recovered by pipette. The aqEtOH extracts were combined and dried by rotary evaporation (∼1 h; 35 °C) to recover the lipid fraction (*SI Appendix*, Fig. S13). Cellular material remaining in the pellet was suspended in 2.5 mL of 1 M aqNaOH and gently agitated for 1 h to hydrolyze RNA. The hydrolysis was quenched by immersing the sample tube in an ice bath for 15 min, after which the sample was acidified to pH 1 by adding 2.5 mL of 1 M aqHCl and 0.5 mL of 50% aqTCA. The mixture was allowed to rest at room temperature for 15 min to precipitate proteins and DNA. The suspension was centrifuged (12,100 rcf; 30 min at 4 °C), and the supernatant containing the RNA was removed by pipette and dried by rotary evaporation (∼1 h; 35 °C). The remaining pellet was suspended twice with 5 mL of 5% aqTCA then twice with 5 mL of 95% aqEtOH and centrifuged after each rinse (12,100 rcf; 30 min at 4 °C). The remaining solids were suspended in 5 mL of 5% aqTCA then immersed in boiling water for 30 min to hydrolyze the DNA. Immediately after, the suspension was centrifuged (12,100 rcf; 30 min at 4 °C) and the DNA-containing supernatant recovered by pipette (*SI Appendix*, Fig. S13). The residual pellet was suspended twice with 5 mL of ice-cold 5% aqTCA then twice with ice-cold 95% aqEtOH, centrifuging after each wash (12,100 rcf; 30 min at 4 °C). Finally, the pellet was hydrolyzed to recover proteins by treating the remaining cell pellet with 5 mL of 1 M aqNaOH (37 °C, 18 h). A small amount of insoluble debris remained after the protein extraction. This was removed by centrifugation (12,100 rcf; 30 min at 4 °C) and syringe filtration of the supernatant.

### Protein Extraction and Precipitation.

We fractionated the peptides and denatured “soluble” proteins from native HMW “insoluble” proteins using the protocol described by Hutchins et al. (129). We lysed the cell pellet from 0.5 L of culture (15 min, room temperature [RT]) with 1 mL of 1% sodium dodecyl sulfate (SDS) extraction buffer (1% SDS, 0.1 M Tris/HCl pH 7.5, and 10 mM ethylenediaminetetraacetic acid (EDTA) then heated at 95 °C for 10 min. We allowed the samples to cool to RT and then agitated them at 350 rpm for 1 h. We centrifuged the resulting suspension (20 min, 14,100 rcf) and decanted the supernatant. We concentrated the proteins from the supernatant by membrane centrifugation using 5K molecular weight cutoff Vivaspin units of 6 mL (Sartorius Stedim, Goettingen, Germany). We recovered the retentate (∼300 µL) and added 1 mL of cold 50/50 methanol/acetone solution (acidified with HCl to a final concentration of 0.5 mM). After letting the solution rest for 3 d at −20 °C, we pelleted the insoluble proteins by centrifuge (14,100 rcf for 30 min at 4 °C) and decanted the supernatant.

## Supplementary Material

Supplementary File

Supplementary File

Supplementary File

Supplementary File

## Data Availability

The datasets and computer code supporting the findings in this study are available from Zenodo at https://doi.org/10.5281/zenodo.5117752. The entire MARMICRODB dataset including a comprehensive description, peptide sequences , Kaiju version 1.6.0 (104) formatted databases, scripts, and instructions for how to use the resource is available from Zenodo at https://doi.org/10.5281/zenodo.3520509. GEOTRACES chemical data were processed and matched to metagenome samples using code/methods available fromZenodo at https://doi.org/10.5281/zenodo.3689249. Tara Oceans chemical and hydrographic data were processed and matched to metagenome samples using code/methods available from Zenodo at https://doi.org/10.5281/zenodo.3786232. The list of *Prochlorococcus* core gene families and a sequence profile database are available from Zenodo at https://doi.org/10.5281/zenodo.3719132.
