# Supplementary material for: Phosphonate production by marine microbes: Exploring new sources and potential function
Source: Proc Natl Acad Sci U S A. 2022 Mar 7;119(11):e2113386119. doi: 10.1073/pnas.2113386119 (PMC8931226; doi:10.1073/pnas.2113386119)
Supplement: Supplementary File [file pnas.2113386119.sapp.pdf]

Supplementary Results

Taxonomic breadth of marine phosphonate producers and consumers

We found phosphonate producers in diverse taxonomic groups including 30 unique families and 11 different phyla (*n* domain=2; *n* phylum=11; *n* class=12; *n* order=25; *n* family=30; *n* genus=40; total genomes=6720). Producers include the Alphaproteobacteria, Gammaproteobacteria, Flavobacteriales, Bacteroidia, Actinobacteria*,* Archaea*,* and Cyanobacteria, and also uncultured groups like SAR202 and the candidate phyla Marinamargulisbacteria and Marinisomatia (Fig. S1). Most producer genomes (> 60%) were assigned to the SAR11 clade and the remaining were evenly distributed amongst low frequency families/orders. The ZD0417 (6%) clade, the OM1 clade (4%) and High-light *Prochlorococcus* (4%) were the next most abundant producer groups.

Compared to producers, phosphonate consumers occurred in fewer taxonomic lineages (Fig. S1). For consumers, we identified 13 unique families and 5 different phyla (*n* domain=1; *n* phylum=5; *n* class=5; *n* order=10; *n* family=13; *n* genus=17; total genomes=6720). Over 90% of consumer cells came from just four taxonomic orders: Cyanobacteria, Pelagibacterales, Rhodobacterales, and AEGEAN-169 (Fig. S1). Thirty percent of phosphonate consumers are from the AEGEAN-169 clade (1), 21% from the *Rhodobacteraceae*, 18% from SAR11 clade Ia.3, 8% from SAR11 clade IV, and 12% were High-light *Prochlorococcus*.

We examined the taxonomic distribution of phosphonate producers and consumers in MARMICRODB (2), a database of over 18000 archaeal, bacterial, eukaryotic, and viral genomes predominantly from the marine environment, but also including terrestrial and host-associated systems. We found phosphonate producers in most of the same marine taxonomic groups as the GORG database (Fig. S2). Unlike GORG, MARMICRODB includes many genomes isolated from the deep ocean (> 500 meters) and from marine sediments. Many deep ocean planktonic groups such as Chloroflexi (including the SAR202 clade) and Marinimicrobia (SAR406) displayed phosphonate production potential. We also found *pepM* and the *ppd* expressed in 24/186 marine transcriptomes from the MMETSP (3) mostly from dinoflagellates as well as in the abundant and cosmopolitan marine Prasinophyte, *Ostreococcus*.

We also searched for phosphonate production genes in Archaea, since past work identified phosphonate production by the Marine Group I archaeon, *Nitrosopumilus maritimus* (4). Compared to bacteria, phosphonate-producing archaea were rare in the euphotic zone (< 3% of all GORG producers), and phosphonate consuming archaea were absent (Fig. S1). However, planktonic archaea comprised a small portion (14%) of GORG-Tropics, with 80% of all archaea from Marine Group II and 20% from Marine Group I. However, 37% of all Marine Group I SAGs contained phosphonate biosynthesis genes. All phosphonate producing MGI archaea were isolated from below 100 meters and included both genera *Nitrosopumilus* (20% of MGI) and *Nitrosopelagicus* (80% of MGI). In MARMICRODB - which had better representation of Archaea - fewer than 1.5% of all archaeal genomes were phosphonate producers (n=18/1256). Thus, archaea may be abundant phosphonate producers at the base of the euphotic zone and mesopelagic, but within the sunlit ocean, other bacterial producers clearly dominate.

Seasonal dependence of phosphonate producers at BATS

We examined the abundance of phosphonate producers in two long-standing ocean time-series: The Bermuda Atlantic Time-series Study or BATS in the North Atlantic Ocean (Sargasso Sea) and the Hawaii Ocean Time-series or HOT near Hawaii in the North Pacific Ocean. Both study sites are in oligotrophic ocean gyres and have comparable biological productivity and carbon export (5). *Prochlorococcus* and SAR11 are the most abundant planktonic cells in the upper 300 meters at both sites (6), but the two sites have different seasonal forcings. BATS experiences strong convective mixing during the winter months, and HOT is more stably stratified, although the latter does display some seasonal productivity (7). The two sites also differ in their biogeochemical inventories. Inorganic phosphate concentrations are lower at BATS than HOT because of differential iron supplies (8); atmospheric iron fluxes are higher at BATS compared with HOT (9, 10) which favors dinitrogen fixation and drives phosphate concentrations to lower values. The selection pressures due to differences in total dissolved P inventories between HOT and BATS are reflected in the gene content variation of their *Prochlorococcus* populations: Those in the Sargasso Sea are more likely to have additional P acquisition genes such as alkaline phosphatase (11,

12).

Phosphonate producers had similar abundances at both HOT and BATS and in other samples from the global ocean. For SAR11 and total bacterioplankton these abundances generally increased with depth and were highest below the subsurface deep chlorophyll maximum layer (> 200 meters). *Prochlorococcus* phosphonate producers were at highest abundance in the surface layer (approximately 10 meters) at HOT and BATS and decreased in abundance with depth. In the surface layer at HOT, approximately 6-9% of *Prochlorococcus* genomes, 10% of SAR11 genomes, and 10% of total bacterioplankton genomes had a phosphonate production gene cluster. At BATS, a significant seasonal effect on the abundance of all phosphonate producers in the surface layer that temporally corresponds with the recurring deepening of the mixed layer during winter months was observed (Fig. S8). For example, *Prochlorococcus* phosphonate producers are most abundant from February to May then decline to negligible levels in the summer at the onset of peak water column restratification. The seasonality of SAR11 producers and the total community producers was tightly coupled, suggesting that peak producer abundance occurs during periods with the deepest mixed layer (November through the following March). This pattern follows the seasonal succession of ecotypes at BATS where SAR11 clades IIa and IIb are most abundant in the upper 200 meters during the winter (13). SAR11 clades IIa and IIb were also the most abundant producers in GORG-tropics and GORG-BATS after the surface clade Ia, and SAR11 IIb abundance showed a strong positive correlation with phosphonate producers in the global dataset.

Biotic and abiotic covariates shaping the global distribution of phosphonate producers

For *Prochlorococcus* producers we find that a small, but significant, amount of variation is explained by phosphate concentrations, and the modeled climatological mean of dissolved organic phosphorus (Fig. S6, Fig. S9). The models suggest that on average *Prochlorococcus* phosphonate producers are most abundant where phosphate and DOP concentrations are highest. For example, the North Atlantic and Mediterranean had the lowest median abundance of *Prochlorococcus* producers. In the deeply sequenced North Atlantic sample GORG-BATS we detected no *Prochlorococcus* phosphonate producer genomes and significantly more *Prochlorococcus* phosphonate consumer genomes than in the rest of the global samples. This pattern mirrors the overall depletion of phosphate relative to other nutrients in the North Atlantic (8). We interpret this as reflecting potential selection against the phosphonate biosynthesis trait in *Prochlorococcus* populations from consistently P-limited regions, such as the tropical North Atlantic, due to the additional P cost of phosphonate biosynthesis. The largest proportion of variation for *Prochlorococcus* phosphonate producers is explained by the total abundance of *Prochlorococcus.* That is, *Prochlorococcus* phosphonate producers are more abundant when *Prochlorococcus* constitutes a smaller fraction of the total microbial community. It is unclear what ecological mechanisms might underlie this pattern.

The relative abundances of SAR11 phosphonate producers and total bacterioplankton producers were strongly positively correlated, consistent with SAR11 being the dominant phosphonate producer in GORG-tropics and GORG-BATS. Thus, the relative abundances of SAR11 producers and total producers were shaped by many of the same factors. A small but significant amount of variation in SAR11 phosphonate producers was explained by increasing climatological mean nitrate concentrations and dissolved aluminum concentrations. This reflects that the highest average abundance of SAR11 producers occurred in the North Atlantic and Mediterranean Sea, regions which have higher than average dissolved aluminum concentrations (14) and climatological mean nitrate concentrations in the upper 100 meters. Generally, we find that the greatest amount of phosphonate producer variation is explained by multiple depth-dependent biotic factors implying that increasing depth is a unifying master parameter. Total bacterial/archaeal and SAR11 phosphonate producers increase alongside depth-associated biotic factors including low light *Prochlorococcus* and the abundance of mesopelagic groups like Archaea (15) and SAR11 IIb (16). Phosphonate producers are negatively correlated with known surface clades like SARII IV and V and abiotic factors like dissolved manganese concentrations (manganese being a surface enriched element (17)). These modeling results offer a prediction that bulk phosphonate concentration is greatest in the lower reaches of the euphotic zone and upper reaches of the mesopelagic. Future chemical studies targeting the 100-300 meters depth range will hopefully provide greater understanding of phosphonate cycling in the upper ocean.

Estimation of the global phosphonate production by MGI Thaumarchaeota, *Trichodesmium*, *Prochlorococcus,* and SAR11

In the surface oligotrophic ocean - where *Prochlorococcus* thrives - phosphonates constitute about 20% of the HMWDOP pool (18) which itself is ~ 25-30% of the DOP pool (based on carbon measurements) (19). Assuming a mean concentration of 200 nM for DOP in the surface of oligotrophic areas (20) then HMWDOP and phosphonate concentrations are 50 nM and 10 nM respectively. With a phosphonate residence time estimated to be between 1-2 years (we used a mean value of 1.5 years) (21, 22) this corresponds to a global phosphonate production rate of 6.7 nM y^{-1}.

MGI Thaumarchaeota generally occur at low abundance in the euphotic zone (23) but rapidly increase in abundance in the upper mesopelagic where they can account for nearly 40% of all DNA staining bacterioplankton (22-24). Since our interest is the euphotic of the oligotrophic gyres we assume a mean MGI Thaumarchaeota abundance in this depth range (upper 200 meters) of 5×10^4 cells L^{-1} (24–26). Assuming a volume of 1.10×10^{19} L for the upper 200 m of the oligotrophic gyres with the addition of the Mediterranean Sea (27) and that 40% of MGI Thaumarchaeota cells are phosphonate producers (this study) results in 2.20×10^{23} phosphonate producing cells. With a cellular P quota of 4.84×10^{-17} mol P $cell^{-1}$ (28), the standing P reservoir in phosphonate producing MGI Thaumarchaeota is 1.07×10^7 mol P. Assuming a growth rate for open ocean Thaumarchaeota of 0.1 d^{-1} (28, 29) and that 25% of cellular P is in the form of phosphonates (29), global annual phosphonate production for MGI can then be calculated as 0.009 nM y^{-1} which would account for 0.15% of the total phosphonate production in the euphotic zone of the gyres. For additional context, the entire cellular P production rate of MGI in the euphotic zone would account for approximately 0.65% of the annual phosphonate budget. Thus, MGI alone cannot account for the observed phosphonate concentrations in the euphotic zone of the oligotrophic gyres. However, MGI are likely important phosphonate producers in the mesopelagic zone where their abundance becomes substantially higher.

We also performed a similar calculation for *Trichodesmium* following the calculations from Dyhrman (30). The raft morphology of *Trichodesmium* can occur at densities of 60 colonies m^{-3} in non-bloom conditions (31). Extrapolating from phosphonate measurements from *Trichodesmium* in culture and in the field (30) this results in a standing stocking stock of 0.013 mol L^{-1} of phosphonate in *Trichodesmium*. This is a very coarse estimate because there is high variability in P content per colony and colony abundance across the global tropical and subtropical gyres. Furthermore, these numbers are parameterized from the western North Atlantic Ocean which may not be representative of other regions. Assuming a specific growth rate of 0.25 d^{-1}, which is the maximum observed in culture (33), this would result in an annual production of 1.19 nM y^{-1} or ~17% of the global average phosphonate production rate. Likewise, assuming that *Trichodesmium* fixes 60 Tg new N y^{-1} across the ocean (34), that fixed N is its sole N source, and that the N:P ratio of *Trichodesmium* is 30 (35) would result in 1.43 $\times10^{11}$ mol P y^{-1} production. If 10% of cellular P exists in the form of phosphonate then this would account for about 19% of the observed phosphonate budget in the gyres.

Prochlorococcus is responsible for fixing 4 GT C y^{-1} which represents 0.33 x 10^{15} mol C y^{-1} (35). We identified about 6% of *Prochlorococcus* as putative phosphonate producers. Since we found no relationship between clade and *pepM* we assume that this group behaves like *Prochlorococcus* SB with regard to phosphonate and like an average *Prochlorococcus* with regards to all the other metabolisms. Therefore, the phosphonate producer strains are responsible for the fixation of 0.02 x 10^{15} mol C y^{-1}. Assuming all *Prochlorococcus* phosphonate producers have a similar C/P ratio than *Prochlorococcus* SB i.e. C/P = 156/1 (36) then they fix 12.8 x 10^{10} mol P y^{-1}. With 40% of the P allocated to phosphonates (our study) this yields a production of 5.1 x 10^{10} mol Phn y^{-1}. As *Prochlorococcus* inhabits the top 200 m of oligotrophic gyres, *Prochlorococcus* could produce 4.6 nM Phn y^{-1} sustaining ~69% of the phosphonate yearly production. We can also estimate the contribution to the phosphonate production from field measurements. At a mean concentration of *Prochlorococcus* of 4.4×10^7 cells L^{-1} in the euphotic zone between 30N and 30S (37), assuming that 6% of *Prochlorococcus* cells are phosphonate producers with a cellular P quota of 4.2×10^{-18} mol $cell^{-1}$ (observed in natural populations from equatorial North Atlantic with C:P ratio = 200-800) (38), and a specific growth rate of 0.4 d^{-1} (39) we can calculate that

Prochlorococcus accounts for 10% of the phosphonate budget in the euphotic zone of the oligotrophic gyres. Thus, it is plausible that *Prochlorococcus* could account for between 10% to 70% of the global euphotic zone phosphonate production.

A similar calculation can be done for SAR11. SAR11 has an estimated number of cells in the global ocean of 5×10^{8} cells L^{-1} (40) or ~5.5×10^{27} cells in the top 200 m of the oligotrophic ocean where SAR11 is most abundant (41). Based on our study, 18% or 9.9×10^{26} cells of SAR11 that are putative phosphonate producers. The P quota of SAR11 growing exponentially in culture, is 0.16×10^{-16} mol P $cell^{-1}$ (42). With a specific growth rate of 0.1 d^{-1}, SAR11 phosphonate producers are responsible for the yearly production of 57.8×10^{10} mol P y^{-1}. We do not have an experimental estimate of the amount of P allocated to phosphonate production in SAR11. However, even with a phosphonate allocation of only 5% SAR11 could produce 2.9×10^{10} mol Phn y^{-1} which corresponds to a production of 2.6 nM Phn y^{-1} and could sustain ~40% of the yearly phosphonate production. With a phosphonate cellular abundance of 10%, comparable to *Trichodesmium erythraeum*, SAR11 could sustain ~80% of the yearly phosphonate production. Using data from cells collected from the tropical North Atlantic Ocean where SAR11 has a mean cellular P quota of 0.065×10^{-16} mol P $cell^{-1}$ (43), a specific growth rate of 0.1 d^{-1} (44) and a standing stock of 5×10^{8} cells L^{-1} (44) we estimate that SAR11 accounts for ~15% of the euphotic zone phosphonate production if 5% of its cellular P is allocated to phosphonates.

Evidence for phosphonylated surface layer structures by *Prochlorococcus* SB

The phosphonate biosynthesis gene cluster contains genes predicted to be involved in cell wall/membrane/envelope biogenesis and extracellular polysaccharide biosynthetic processes (Fig. S5), suggesting that *Prochlorococcus* SB may produce extracellular saccharides modified with phosphonate groups. We also found that genes coding for glycosylation reactions were statistically more abundant within ± 5 Kbp of the phosphonate biosynthesis cluster than in the remainder of the complete genomes from *Prochlorococcus* SB and *Pelagibacter* sp. HTCC7217 and RS40 (Table S5). Many of these genes encode glycosylation enzymes involved in cell membrane/envelope biogenesis (Fig. S5). However, our chemical data shows that phosphonates are associated with a high molecular weight protein fraction. We propose that, in *Prochlorococcus* SB, phosphonates serve as molecular decorations of glycan chains bound to high molecular membrane-anchored proteins. In glycoproteins sugar polymer chains are covalently attached to amino acid side-chains of a parent protein through post-translational modification.

Glycoproteins were once thought to be exclusive to eukaryotes, but recent efforts indicate that they are ubiquitous in bacteria and archaea (45). In bacteria glycans are attached to specific target proteins via both N- and O-linked glycosylation (46). Protein substrates for glycosylation reactions include the motor protein flagellins, pilins found in bacterial pilus structures (46), and S-layers which are two-dimensional paracrystalline structures that cover the outer surface of bacteria and archaea (47). Protein glycosylation occurs via two distinct mechanisms: the direct attachment of glycan monomers or small chains to proteins via glycosyltransferases (common in flagellins and adhesins) and the pre assembly of glycan onto lipid carriers followed by transfer en masse to a protein acceptor (48). Recently, it has been discovered that many bacteria also possess general O-linked glycosylation systems capable of promiscuous modification of numerous different types of membrane associated proteins (49–51).

In *Prochlorococcus* SB, we find genes with homologies to both S-layer biosynthesis and the general O-linked glycosylation system. S-layer biosynthetic clusters encode genes for both biosynthesis and export of lipopolysaccharide anchors (47) and the biosynthesis and export of an extracellular glycoprotein containing the critical Ca^{2+}- binding hemolysin domain (52, 53). In the *Prochlorococcus* SB genome between 1.15 - 1.17 Mbp we find genes encoding putative surface antigens of the filamentous hemagglutinin-glycoprotein family, Ca^{2+}- binding hemolysin domain proteins, and a type-I secretion system ABC transporter, and other antigen-polysaccharide repeats (Table S2). Most compelling, however, is the presence of general O-linked glycosylation reaction enzymes adjacent to the *Prochlorococcus* SB, *Pelagibacter* HTCC7217 and *Pelagibacter* RS40 phosphonate biosynthesis clusters (Fig. S5). In *Prochlorococcus* SB we find homologs to known genes involved in extracellular capsule synthesis/export, glycan chain extension reactions, and glycosyl transferase reactions. The presence of these capsular biosynthesis enzymes is consistent with the significant cross-talk between lipopolysaccharide biosynthesis and glycoprotein biosynthesis (48) and the shared glycan substrates and enzymes between both pathways (54). Most importantly we identify an initiating glycotransferase (PglC)

which links the first glycan subunit to a lipid carrier, a flippase (Wzx) which translocates the assembled glycan-bound lipid carrier to the periplasmic face, and an O-oligosaccharyltransferase (PglL) which is the critical enzyme moving the assembled glycan chain to the final acceptor protein (54, 55). The striking mutual exclusivity we observe between phosphonate production and consumption genes in marine genomes may also be related to the molecular mechanisms and subcellular location of the machinery from both pathways. For example, both the catalytic domains of C-P lyase (56) and the assembly of glycans (45) occur on the cytoplasmic side of the bacterial inner membrane. A cell simultaneously expressing both these systems might cannibalize its own phosphonates. Taken together our findings strongly suggest that *Prochlorococcus* SB has the genomic potential to produce surface-expressed phosphonoglycoproteins.

Detailed Methods

Genomic data sources:

We used two collections of genomes for the comparative genomics analysis: MARMICRODB (56) (https://zenodo.org/record/3520509) and the Global Ocean Reference Genomes (GORG) Tropics dataset (57). MARMICRODB contains over 18000 archaeal, bacterial, eukaryotic, and viral genomes from predominantly the marine environment, but also terrestrial and host-associated systems. GORG Tropics consists of approximately 13000 single-cell genomes sequenced from 28 samples across the tropical surface ocean. Roughly 6000 genomes from GORG Tropics come from a single sample (GORG BATS) from the Sargasso Sea. For quantitative analysis of the complete GORG dataset we randomly subsampled GORG BATS (Sample SWC-09) genomes to the median genomes per sample (N=241) from the remainder of GORG Tropics.

Homology searches:

We identified phosphonate biosynthesis and catabolism proteins by homology to a collection of hidden Markov models using HMMERv3.1b2 (http://hmmer.org/) and the trusted cutoffs of each individual model. *pepM* peptide sequences were initially identified using PF13714 (PEP_mutase) and then aligned to the Tigrfam (58) Hidden Markov Model (HMM) TIGR02320 using hmmalign. We identified authentic *pepM* peptide sequences by strict presence of conserved active site motif "EDK(X)5NS" or if they lacked at most two active site residues but were located adjacent (within 5000 nucleotides upstream/downstream) to a genes coding for *ppd* (TIGR03297) or *mpnS* (59–62). The PF13714 model identified many sequences lacking the PepM active site motif, and with significant similarity to the related isocitrate lyase superfamily PF00463. Sequences passing the PF13714 bitscore cutoffs, but lacking the "EDK(X)5NS" motif were classified as members of the isocitrate lyase superfamily. We identified peptide sequences from authentic *mpnS* and the related *hepDI/hepDII* genes using custom HMMs built from alignments of curated sequences identified as containing essential catalytic residues (63). We constructed these alignments using MAFFT v7.273 (64) in E-INS-i iterative refinement mode. We trimmed the alignment to peptide positions 317-792 to isolate the core catalytic residues and used to construct HMMs. We identified *mpnS*, *hepDI*, and *hepDII* sequences in MARMICRODB and GORG-tropics using the resulting HMMs (evalue < 0.05), and aligned the resulting sequences using MAFFT. We manually inspected the alignments to remove sequences lacking the essential catalytic residues and realigned them iteratively after we removed poorly-aligned sequences.

For phosphonate catabolism proteins, we inspected gene neighborhoods to confirm the presence of multiple co-occurring genes from the PhnYZ (65), C-P lyase, 2-aminoethylphosphonate, and phosphonoacetaldehyde phosphonatase catabolism pathways (66). Specifically, valid C-P lyase clusters required at least five other genes from *phnC*, *phnD*, *phnE*, *phnF*, *phn*G, *phnH*, *phnI*, *phnJ*, *phnK*, *phnL*, *phnM*, *phnN*, *phnO*, *phnP* to occur within uninterrupted i.e. no scaffold/contig breaks, 16000 nucleotide sliding windows. Valid phosphite clusters required two of *ptxD*, *phnY*, and *phnZ*. Valid 2-aminoethylphosphonate clusters required co-occurring *phnX* and *phnW* or *phnW*, *phnA*, and *phnY*. Valid phosphonoacetaldehyde phosphonatase clusters required *phnZ*, *hpnW*, and *hpnZ*. All catabolic pathways other than C-P lyase required genes to co-occur within uninterrupted 10000 nucleotide sliding windows.

We also identified two sets of ten highly conserved, single-copy families from *Prochlorococcus* and SAR11 lineages for use in normalization with metagenome profiling. These gene families had the ten lowest dN/dS ratios of the entire pangenome, that is the highest purifying/stabilizing selection, for *Prochlorococcus* or SAR11, and should thus be highly specific and sensitive for metagenomic quantification. *Prochlorococcus* and SAR11 pangenomes were characterized and defined using PanX (67). To profile metagenomes for total bacterioplankton, we used fetchMG from the mOTUs2 tool (68). Briefly, we identified ten COG families (COG0012, COG0016, COG0018, COG0172, COG0215, COG0495, COG0525, COG0533, COG0541, COG0552) from all bacterial and archaeal genomes in MARMICRODB. To reduce redundancy, we clustered protein sequences from each COG at 90% sequence identity using MMseqs2 v4e23d (69) and used the 90% clustered sequence representatives in metagenome search databases. The HMM protein sequence profiles, results from these searches, and descriptions of the sequence families are available from https://github.com/slhogle/phosphonates.

Multiple sequence alignments, phylogenetic inference, and topological comparison:
We aligned authentic *pepM* peptide sequences containing the "EDK(X)5NS" motif to TIGR02320 using hmmalign and trimmed using trimAl v1.4.rev22 (70) with the automated -gappyout option, and we manually inspected the alignments to ensure the veracity of the "EDK(X)5NS" motif. We created the multi-phylum genome phylogenies from genome assemblies with confirmed phosphonate biosynthesis pathway or at least one confirmed phosphonate catabolic pathway. We used the GTDB-Tk v1.3.0 pipeline (71) with default settings and the GTDB R05-RS95 database (72) to identify conserved proteins (120 bacterial proteins/122 archaeal proteins) and generate concatenated multi-protein alignments. We filtered alignment columns using the bacterial and archaeal alignment masks from (http://gtdb.ecogenomic.org/downloads). We then removed columns represented by fewer than 50% of all taxa and/or columns with no single amino acid residue occurring at a frequency greater than 25%. We further trimmed the alignments using trimAl with the automated -gappyout option to trim columns based on their gap distribution. The multi-phylum genome phylogenies and the PepM phylogeny were inferred using FastTree v2.1.10 (73) under the GAMMA model of rate heterogeneity and the WAG+ substitution model (74). Support values were determined using 100 non-parametric bootstrap replicates. Both the PepM tree and genome tree were left unrooted. Phylogenies and associated data were visualized using ggtree (75).

Gene enrichment analysis:
We annotated the 16 *Prochlorococcus* and 22 SAR11/*Pelagibacterales* genomes containing verified *pepM* sequences with eggNOG 4.5.1 (76) using eggNOG-Mapper v1.0.3-3-g3e22728 (89). We then collected the resulting KEGG orthology annotations (77) from all genes and tested for enrichment of modules and pathways in the KEGG hierarchy in genes within 10000 nucleotides upstream/downstream of the *pepM* sequence start and stop coordinates respectively. Significant enrichment of KEGG categories were determined using the hypergeometric test (78) implemented in clusterProfiler v3.8 (79). We excluded genomes from the analysis that had contig/scaffold breaks within 10000 nucleotides upstream/downstream of the *pepM* sequence.

Identification of *pepM* sequences within *Prochlorococcus* genomic islands:
We predicted genomic islands using Hidden Markov Models trained from conserved gene synteny patterns in closed *Prochlorococcus* genomes as described before (80). Briefly, we assume genomic islands to be contiguous stretches of DNA enriched in flexible genes families (i.e. genes found in a subset of all genomes). We then used previously described genomic islands in six *Prochlorococcus* genomes (81) to define core, flexible, and inconclusive gene "states" in four distinct hidden markov models, each with two hidden states (island or non-island). We then used empirical gene family frequencies as a proxy for the core/flexible/inconclusive state of each gene and the gene order on each scaffold as input to the Viterbi algorithm for predicting the hidden island state of each gene in all other *Prochlorococcus* genomes.

Metagenome read classification:
We created Diamond v0.9.22.123 (82) search databases for each of the three ten-gene marker gene sets identified for *Prochlorococcus*, SAR11, and all bacterioplankton using reference genomes from MARMICRODB. We also created Diamond databases for only *Prochlorococcus pepM*, only SAR11

pepM, and all bacterial and archaeal *pepM* peptide sequences identified with the "EDK(X)5NS" catalytic motif from MARMICRODB only. We identified the marker gene sets using a two-tiered search strategy using Diamond in default fast mode. We first searched the metagenomes against reduced marker sets clustered at 70% amino acid identity by MMseqs2. We pulled all reads with hits to these reduced marker sets, then searched the reads against the entire MARMICRODB, and finally retained read mappings with a best scoring match to a MARMICRODB protein from the original marker family. We searched metagenomic reads against the *pepM* databases using Diamond (mode --more-sensitive) and score cutoffs of 55% amino acid identity and an E-value of 1e-5. We determined these cutoffs empirically to be those that produced the highest F Score (harmonic mean of precision and recall) from mock metagenomes simulated from GORG-tropics (see next section, Fig. S11). Since the E-value is a function of database size it is important to note that the significance of this cutoff is specific to the reference databases here. In the case of ambiguous alignments (i.e. identical best alignment scores to sequences from different taxonomic groups) we classified reads using a probability-weighted random sampling of all taxonomic groups matching the best hits. We derived the probability distributions for random samplings from the taxonomic classification uniquely mapped *pepM* reads.

To normalize *pepM* reads across metagenomes, we calculated the length normalized counts (RPKM) of the 10 single copy marker genes from *Prochlorococcus*, SAR11, and all bacteria and archaea in each metagenome as reads per kilobase marker and then estimated the number of 'genome equivalents' in each metagenome as the median of the 10 marker families. We also calculated the length normalized abundance of *pepM* from *Prochlorococcus*, SAR11, and all bacteria and archaea as reads per kilobase from each metagenome. We then divided the length normalized abundance of *pepM* by the estimated number of genome equivalents for each taxonomic group to estimate the fraction of genomes with phosphonate biosynthesis potential. To ensure robust estimates of normalized abundance, we excluded samples where the median marker gene coverage was less than 100X for all taxonomic groups.

Quantification of *pepM* and marker genes using short metagenomic reads:
Metagenomic classification of diverse functional genes can present methodological challenges because the amount of discriminative taxonomic signal in these genes may be marginal or confined to specific regions of the gene. For example, *pepM* appears to be horizontally transferred both within closely related microbial groups like *Prochlorococcus* and between diverse microbial lineages. In some cases, it may be difficult to taxonomically assign a short metagenome read derived from a *pepM* gene because the read may map equally well to a *pepM* sequence from multiple taxonomic lineages. We quantified the skill with which we could classify *pepM* sequences derived from SAR11, *Prochlorococcus*, and all bacteria and archaea genomes using mock metagenomes of known composition.

We simulated mock metagenomes from 200 randomly sampled single cell template genomes from the approximately 13000 genomes from GORG-tropics. The 200 template genomes were sampled from all available taxonomic orders but were restricted to those genomes from each order that fell into the top 10% highest estimated completeness from checkM. Genomes estimated to be below 30% completeness were excluded. These criteria resulted in 74 SAR11 and 48 *Prochlorococcus* template genomes. We simulated 150 basepair paired-end Illumina sequencing reads mock metagenomes using randomreads.sh from BBMap v37.90 (https://sourceforge.net/projects/bbmap/) with 3x coverage, an insert distribution of 155-320 bp and parameters snprate=0.005, insrate=0.0005, delrate=0.0005, and subrate=0.0005. We next included all identified *pepM* sequences from GORG-tropics to represent the full diversity of *pepM* sequences in the surface ocean. As a decoy case we also included all GORG genes from the isocitrate-lyase superfamily, which have superficial similarity to *pepM* but lack the essential conserved active site motif "EDK(X)5NS." We then cut these authentic and decoy *pepM* gene sequences with 75 bp upstream and downstream flanking DNA sequence from their parent genomes and simulated sequencing reads using the same parameters for the full genomes but with a sequencing depth of 30x. This step produced sequencing reads derived from 25, 96, and 14 authentic *pepM* sequences from *Prochlorococcus*, SAR11, and all remaining bacteria and archaea, respectively. It also generated reads from 49 and 134 isocitrate lyase decoy sequences from bacteria and archaea and SAR11, respectively. The final resulting simulated metagenomic reads were preprocessed and merged as the Tara ocean and GEOTRACES metagenomes.

Because we knew the precise quantity of *pepM*-derived reads in the simulated metagenome we could then iteratively calculate the true positive rate, true negative rate, positive predictive value, and the F1 score (harmonic mean of the true positive rate and positive predictive value) for different combinations of sequence similarity cutoffs. In this case the positive predictive value is the probability that a metagenomic read classified as *pepM* is derived from a *pepM* sequence, the true positive rate is the percentage of true *pepM* reads correctly identified as such, and the true negative rate is the percentage of false *pepM* reads correctly identified. We selected the percent identity and E value classification cutoffs that produced the highest F1 score for *pepM*. Ultimately, we selected an E value cutoff of 1e-5 and a percent identity cutoff of 55%, which resulted in a true positive rate of 95% and a true negative rate of 99% for classifying any bacterioplankton *pepM* (Fig. S11). For *Prochlorococcus pepM* and SAR11 *pepM* sequences, respectively, our classification criteria produced a true positive rate of 96%/60% and a true negative rate of 97%/98%. These performance metrics suggest that, in all cases, we are unlikely to classify superficially related reads as a true *pepM* sequence. For *Prochlorococcus* and combined bacteria/archaea it appears that we correctly capture 95-96% of the total *pepM* sequence diversity in the mock metagenomes. The true positive rate for SAR11 was significantly lower (60%) which suggests that 40% of SAR11 *pepM* sequences are missed and probably assigned only to the bacteria/archaea domain level. This result is consistent with the high sequence similarity between a handful of *pepM* genes from SAR11 and other diverse bacterial groups, and suggests that our metagenomic database probably underestimates the contribution of SAR11 to the total inventory of *pepM* genes in the ocean. For example, the abundance discrepancy between GORG-tropics genomes and the metagenomes for SAR11 *pepM* is likely explained by the low estimated sensitivity (60%) for our method to SAR11. Assuming we missed 40% of SAR11 *pepM* sequences, the corrected percentage of SAR11 genomes with *pepM* estimated from metagenomes is 17% which agrees very well with the independent results from GORG-tropics (18%) (Table S1). The misclassification of SAR11 *pepM* to other groups probably also accounts for some of the significant positive correlation between SAR11 and combined bacteria/archaea *pepM* abundance (Fig. S9). Overall, these classification metrics indicate that we are unlikely to misclassify *pepM* reads from marine metagenomes and suggest our relative abundance estimates are robust.

Biotic and abiotic data associated with metagenomes:
We obtained and preprocessed the biotic and abiotic variables used in this study as described in detail here: https://doi.org/10.5281/zenodo.3689249 and here: https://doi.org/10.5281/zenodo.3786232. We obtained inorganic phosphate concentrations from the GEOTRACES Intermediate Data Product IDP2017 version 3 (83), specifically from sections GA02 (84, 85), GA03, GA10(86), and GP13. We obtained inorganic phosphate and DOP concentrations from the Tara Oceans project (87) (https://doi.pangaea.de/10.1594/PANGAEA.875579). We obtained modeled climatological dissolved organic phosphorus and other variables from the MIT Darwin model (v0.1_llc90, http://darwinproject.mit.edu/) from the Simons Collaborative Marine Atlas project (88) using pycmap v0.1.2 (https://doi.org/10.5281/zenodo.3561147). We generated taxonomic profiles derived from metagenomic reads as described earlier (88) using the MARMICRODB database. As a first order estimate of *Prochlorococcus* and SAR11 ecotype relative abundance we divided the number of reads mapping to each ecotype by the number of reads mapping in total the *Prochlorococcus* genus or the family *Pelagibacterales* for SAR11. Some proportion of reads mapping to highly conserved (core) regions can only be reliably classified at the genus or family level thus our ecotype relative abundance estimates using total read counts are underestimated.

Random Forest Regression:
We used random forest regression, a nonparametric approach, to identify biotic and abiotic factors structuring the distributions of phosphonate producers. We selected random forest regression because the dataset has nearly 45 abiotic/biotic variables, many of which are correlated, and random forest is capable of handling many predictor variables and is robust to covariate co-linearities. We trained one random forest model (n_{trees}= 1000) for each taxonomic group; *Prochlorococcus*, SAR11, and bacteria plus archaea. Each model was trained using up to 44 abiotic/biotic variables including trace metal and macronutrient data from GEOTRACES and Tara Oceans, modeled climatological means from the MIT Darwin model (http://darwinproject.mit.edu/), and ecotype relative abundances. For the outcome variable (normalized *pepM* relative abundance) we used a modified splitting rule for tree construction that maximized the log-likelihood of the beta distribution on the interval [0,1] (89). Although our relative

abundance measure is not theoretically restricted to the unit interval (values greater than one could exist, e.g. if the *pepM* copies per genome greatly exceeded one), in practice *pepM* relative abundance was always bounded [0,1] in our datasets. We performed random forest regression using nested ten-fold cross-validation to prevent data leakage to the validation phase (90). During the cross-validation and tuning phase, we performed feature selection heuristics to find the top 50th percentile of informative variables for *Prochlorococcus*, SAR11, and combined bacteria/archaea. We reserved 20% of the data for estimating final model performance. The remaining 80% of training data was split into 10 resampled partitions, each with analysis and assessment partitions, to tune and estimate the performance of preprocessing, supervised feature selection, hyperparameter tuning steps. We tuned hyperparameters (mtry, min.node.size), by maximizing the coefficient of determination from correlation (model R^2) and minimizing the root mean square error (RMSE). We used CAR Scores (91) for recursive feature elimination to retain only the top 50th percentile of informative variables. We included the feature elimination step to reduce the computation costs and runtime of the feature importance step (see below). Random forest regression was implemented with the package Ranger (92) and the Beta Forest algorithm (89). For *Prochlorococcus* we ultimately selected 10 model covariates, a minimal node size of 2, and 7 randomly sampled variables at each split resulting in a coefficient of determination (R^2_{Pro}) of 0.74 and Root Mean Square Error ($RMSE_{Pro}$) of 0.04. For SAR11 we selected 15 selected model covariates, a minimal node size of 2, and 6 randomly sampled variables at each split resulting in a coefficient of determination (R^2_{SAR11}) of 0.69 and Root Mean Square Error ($RMSE_{SAR11}$) of 0.03. For the combined bacteria/archaea we selected 21 selected model covariates, a minimal node size of 2, and 8 randomly sampled variables at each split resulting in a coefficient of determination ($R^2_{combined}$) of 0.94 and Root Mean Square Error ($RMSE_{combined}$) of 0.02. For *Prochlorococcus* and SAR11 there was an appreciable amount of variation in producers that the models could not capture (R^2_{Pro}= 0.74, R^2_{SAR11}= 0.69).

We determined predictor variable rankings on the final model from the cross validation step using the Boruta heuristic (93). This step allowed us to identify all predictor variables that consistently performed better than chance and to compare the importance of each variable to a reference importance level, i.e. random data. The Boruta algorithm is a robust, statistically grounded feature ranking method that aims to identify all variables in a dataset that contain useful information for model predictive outcome. This approach is useful when the variables driving the performance model are of interest and not necessarily the ultimate predictive outcome itself. Boruta performs feature ranking by comparing all variables with randomized versions of themselves in an iterative framework. In addition to variable ranking based on relative scores, Boruta, therefore, provides a measure of the significance of each variable relative to noise. There were 10 and 15 covariates that showed better predictive utility than randomly shuffled data for *Prochlorococcus* and SAR11, respectively (Fig. S7). However, most predictive utility was concentrated into the top 5 variables, while the importance of the other lower ranking variables was in most cases only marginally higher than noise. The five most highly predictive variables for *Prochlorococcus* phosphonate producers (Fig. S7, Fig. S9, Table S2) included inorganic phosphate concentrations, the modeled climatological mean DOP concentrations, the subsurface chlorophyll maximum layer type, and the relative abundance of the *Prochorococcus* LLIV clade. The subsurface chlorophyll maximum layer types 1-4 were characterized by deepening SCML depths (~ 20 to 115 meters) while types 5 and 6 were characterized by high fluorescence throughout the upper 75 meters. The top five variables for SAR11 were the relative abundance of total phosphonate producers in the sample, the climatological mean nitrate concentrations, SAR11 IV relative abundance, SAR11 clade IIb relative abundance, and dissolved aluminum concentrations (Fig. S7, Fig. S9, Table S2). The integrated bacteria/archaea phosphonate producer model performed best ($R^2_{combined}$ = 0.94) and the top five variables (SAR11 clade IIb relative abundance, SAR11 *pepM* relative abundance, archaea relative abundance, SAR11 clade IV relative abundance, Depth) were strongly informative for the random forest prediction when compared to noise. We confirmed these factors for all groups using parametric beta-binomial regression (Table S2)(94).

Beta-Binomial Regression and Generalized Additive Models:
We used the R package corncob (94) and modeled *pepM* relative abundance directly from *pepM* read counts and the median read counts to marker gene sets as "successes/total" which is appropriate for the beta-binomial probability distribution. We used the log-odds link function for both relative abundance and the overdispersion parameter. For each taxonomic group (SAR11/*Prochlorococcus*/bacteria and archaea) we modeled only the top five most important biotic/abiotic variables identified in the Random Forest

regression and variable importance steps. We did not include additional model terms because multiple collinearities between covariates and the additional model complexity prevented model convergence in most cases. We estimated the probability for each biotic/abiotic covariate being informative to the overall model by using bootstrapped likelihood ratio tests (N=1000). We estimated 95% confidence intervals for model coefficients and standard errors using 1000 random draws from the beta-binomial distribution. We estimated seasonal effects in time-series metagenomes using Generalized Additive Mixed Models and Linear Mixed-Effect Models implemented through the mgcv v1.8-26 (95) nlme v3.1-148 libraries in R v3.6.2. To decompose any potential seasonal effects, we fit a cyclic spline term to a variable for the day of the year, which we use as a proxy for season, and we fit a global trend term to the cumulative time since sampling onset. We considered a seasonal effect present if the model term for "day of the year" was statistically significant ($p < 0.05$).

***Prochlorococcus* cultures under P-replete and P-starved conditions:**
To investigate the linkage of *pepM* with phosphonate production, we grew two HLII strains of *Prochlorococcus*: *Prochlorococcus* SB, which has *pepM*, and the closely related *Prochlorococcus* MIT9301, which lacks the *pepM* gene sequence. We grew both strains axenically, under constant light (30 µmol quanta m^{-2} s^{-1}) in artificial seawater medium AMP1 prepared as described before (96), but using 3.75 µM TAPS as a buffer instead of 1 mM HEPES. We first grew *Prochlorococcus* SB in the standard medium i.e. with an inorganic phosphate concentration of 50 µM (N/P = 16/1) to assess whether or not this strain could produce phosphonate under P-replete exponential growth conditions. We then grew them in media with a decreased the inorganic phosphate concentration to 2.28 µM (N/P = 350/1), which did not limit growth during the exponential phase of the cultures, but did result in a P- starved stationary phase culture as the cells ran out of P relative to other nutrients. We verified P-starvation by the initiation of further growth after adding inorganic phosphate to the culture (Fig. 4 inset). For all experiments, we grew biological duplicates to ensure reproducibility. We assessed culture axenicity by flow cytometry and by confirming a lack of turbidity for at least 30 days after inoculation with three test broths: ProAC (97), MPTB (98) and ProMM (96). We cleaned all the glassware and polycarbonate bottles by soaking overnight in 2% detergent (micro), rinsed 6 times with deionized water, soaked overnight in 1 M HCl and rinsed 6 times with ultra-high purity water.

Cell harvest and treatment:
We harvested *Prochlorococcus* SB cultures grown in high N/P medium twice: ~6 L during exponential growth and the remainder (14 L) two days after the onset of stationary phase growth. Culture fluorescence increased in the phosphate-amended cultures, reaching levels regularly observed in *Prochlorococcus* HLII cultures (Fig. 4 inset). We separated the cells from the growth medium by centrifugation (15,970 rcf for 30 minutes at 4°C) and the growth medium was saved for other analyses. We transferred the cell pellets into 50 mL falcon tubes, suspended in Turk Island mix (96) to rinse the cells of external nutrients and centrifuged them (6,523 rcf for 15 minutes at 15°C). We discarded the supernatant and repeated the operation two more times. We flash froze the cells in liquid nitrogen and stored them at -20°C until NMR analyses. We also measured *Prochlorococcus* cell abundance by flow cytometry. We prepared and processed the samples as previously described (99, 100) and ran on a Guava 12HT flow cytometer (Luminex Corp., Austin, TX, USA). Cells were excited with a blue 488 nm laser analyzed for chlorophyll fluorescence (692/40nm), SYBR Green I stained DNA fluorescence content (530/40nm), and size (forward scatter). We ran the samples stained with 1x SYBR Green I (Invitrogen, Grand Island, NY) and unstained then incubated for 60 min in the dark prior to running. All flow cytometry files were analyzed using Guavacyte.

Potential contribution of 1,3,2-dioxaphospholanes to the 18-27 ppm region of the ^{31}P NMR spectrum:
Most cyclic phosphate esters, such as 3´,5´-cyclic nucleotide monophosphates have chemical shifts that fall upfield of acyclic phosphate esters and would therefore not interfere with our interpretation of the *Prochlorococcus* SB NMR spectrum (101). However, some cyclic phosphates, specifically 1,3,2-dioxaphospholanes such as 2´, 3´-cyclic nucleotide monophosphates (2´,3´-cNMP), have ^{31}P chemical shifts that fall within the same spectral region as phosphonates (101,102). 2´,3´-cNMPs are unstable, and are not known to accumulate in cells (102), however to substantiate this we tested two commercially available model compounds for their stability towards mild base hydrolysis; 2´,3´-cyclic adenosine

monophosphate and 2´,3´-cytidine monophosphate. Treatment of these cNMPs with 0.1N NaOH for 2 hours completely hydrolyzed the cyclic phosphate as indicated by an upfield shift of the NMR signal from ~19-20 ppm to ~3-4 ppm (Fig. S13). Treatment of *Prochlocococcus* SB (harvested in stationary phase after exponential growth) with 0.1N NaOH for 2 days, yielded no change in the phosphonate ester/phosphate ester integrals of the spectra (Fig. S13). The pH of the incubation was checked before and after the experiment to ensure basic conditions were maintained throughout. If 2´,3´-cNMPs contributed significantly to the ^{31}P NMR signal at ~ 22 ppm, we would have expected a decrease in the phosphonate/phosphate ester ratio after hydrolysis. This was not observed. Finally, strong base hydrolysis of the Prochlocococcus SB cells (2N KOH, 85°C, 24 hr) yielded 2-hydroxyethylphosphonate as the major product, confirming the synthesis of phosphonates by *Prochlorococcus* SB.

2´,3´-cNMPs are formed by transesterification of nucleotides by the enzymes RNase A (Rnase 1) and binase during RNA depolmerization (103, 104). We found no genes encoding RNase A or binase in the *Prochlorococcus* SB genome. While this analysis does not preclude the formation of 2',3'-cyclic NMPs by other as yet uncharacterized enzymatic pathways, it is consistent with the results of the hydrolysis experiments above that 2´,3´cNMPs do not contribute significantly to the ^{31}P signal at 22 ppm.

Supplementary Tables

Table S1: Relative abundance of phosphonate producers and consumers in GORG single cell genome datasets

Phosphonate	Group	GORG-BATS248	GORG-Tropics	Metagenomes
Consumer	All bacterioplankton	31%	10%	
	Prochlorococcus	64%	14%	
	SAR11	23%	5%	
Producer	All bacterioplankton	9%	15%	15%
	Prochlorococcus	Not detected	6%	6%
	SAR11	15%	18%	10%

Relative abundance estimates are corrected based on the average single cell genome completeness/recovery for each group (see methods). GORG is the Global Ocean Reference Genomes, which is a catalog of single cell genomes isolated from throughout the global tropical surface ocean. GORG-BATS248 is derived from a single 0.4 ml sample collected from the Bermuda Atlantic Time Series in the Sargasso Sea. It is a subset of the larger GORG-Tropics dataset. The metagenomes are from a collection of 644 samples from the upper 250 meters of Tara Oceans and BioGEOTRACES. Phosphonate consumer abundances were not measured in the metagenomes.

Table S2: Model coefficients from beta-binomial regression of PepM abundance

Taxonomic group	Term	Estimate	95% CI [lower]	95% CI [upper]	Std. Error	t value	Bootstrapped LRT P value	LRT P value	Term Significance
Prochlorococcus	*Prochlorococcus* [%]	-0.011	-0.015	-0.007	0.003	-4.433	0.004	4.05E-34	***
Prochlorococcus	Pro LLIV [%]	0.011	0.002	0.018	0.006	1.771	0.077	6.11E-02	.
Prochlorococcus	DOP Clim. avg [µmol kg^{-1}]	1.342	0.360	2.385	0.567	2.366	0.009	4.53E-04	*
Prochlorococcus	PO4 [µmol kg^{-1}]	-0.014	-0.197	0.167	0.126	-0.107	0.745	7.13E-01	NS
Prochlorococcus	DCM type 2	-0.164	-0.304	-0.043	0.077	-2.129	0.003	6.41E-22	*
Prochlorococcus	DCM type 3	-0.561	-0.710	-0.419	0.085	-6.567	0.003	6.41E-22	***
Prochlorococcus	DCM type 4	-0.250	-0.380	-0.125	0.076	-3.277	0.003	6.41E-22	**
Prochlorococcus	DCM type 5	-0.721	-0.970	-0.473	0.134	-5.387	0.003	6.41E-22	***
Prochlorococcus	DCM type 6	-0.084	-0.210	0.037	0.074	-1.136	0.003	6.41E-22	NS
SAR11	Total *pepM* [per genome]	0.021	0.018	0.024	0.002	11.502	0.001	6.94E-29	***
SAR11	NO3 Clim. avg [µmol kg^{-1}]	0.004	0.001	0.006	0.001	2.640	0.120	2.95E-04	**
SAR11	SAR11 IV [%]	-0.014	-0.021	-0.007	0.004	-3.908	0.062	3.34E-05	***
SAR11	SAR11 IIb [%]	-0.005	-0.007	-0.001	0.002	-2.498	0.003	5.99E-05	*
SAR11	Dissolved Aluminum [nmol kg^{-1}]	0.003	0.001	0.004	0.001	3.370	0.003	1.92E-04	***
All bacterioplankton	SAR11 IIb [%]	0.0114	0.0088	0.0137	0.0017	6.835	< 0.001	6.08E-13	***
All bacterioplankton	SAR11 *pepM* [per genome]	0.0452	0.0394	0.0506	0.0035	12.901	< 0.001	8.93E-34	***
All bacterioplankton	Archaea [%]	0.0198	0.0165	0.0223	0.0022	9.083	< 0.001	1.70E-21	***
All bacterioplankton	SAR11 IV [%]	-0.0148	-0.0209	-0.0099	0.0035	-4.237	< 0.001	2.31E-08	***
All bacterioplankton	Pro LLIV [%]	0.0047	0.0031	0.0066	0.0016	2.902	< 0.001	1.88E-07	**
All bacterioplankton	Depth [m]	0.0006	0.0004	0.0008	0.0001	4.117	< 0.001	1.88E-07	***

Coefficient estimates are of unit log-odd change in the probability of sampling a *pepM* read due to a one unit covariate change while controlling for the other covariates. For the categorical variable DCM type the coefficient estimate is from the reference level DCM type 1. 95% confidence intervals are simulated from 1000 random draws from the beta-binomial distribution parameterized from the covariate data. Each covariate is tested for statistical significance using a parametric bootstrapped likelihood ratio test (N=1000) and a classic likelihood ratio (LRT) test using a canonical Chi-squared distribution. Term significance: *** $P < 0.001$, ** $0.001 < P < 0.01$, * $0.01 < P < 0.05$, . $0.05 < P < 0.1$, NS $P > 0.1$. *Prochlorococcus* and Archaea are percentage of all successfully mapped reads to MARMICRODB. Clades LLIV, SAR11 IV, SAR11 IIb are percentage of reads mapped to *Prochlorococcus* and SAR11, respectively. Clim. avg, climatological average from the MIT Darwin model; DCM depth range, deep chlorophyll maximum layer depth category – 1=shallow, 6=deep.

Table S3: Proportion of phosphonate (Phn) and phosphate (Ph) in *Prochlorococcus* SB cells under P-replete and P-starved conditions

Harvest stage	#	Phn proportion	Ph proportion
Prochlorococcus SB P-replete (exponential phase)	1	0.41	0.59
	2	0.43	0.57
	mean	0.42	0.58
Prochlorococcus SB P-starved (stationary phase)	1	0.70	0.30
	2	0.71	0.29
	mean	0.71	0.29

^{31}P-NMR spectra of each growth conditions and each replicates were manually integrated and those values used to calculate the proportion of phosphonate and phosphate in the cells.

Table S4: *Prochlorococcus* SB phosphorus (P), phosphonate (Phn)-P and phosphate (Ph)-P to carbon ratios.

Harvest stage	#	P/C	Phn-P/C	Ph-P/C
Prochlorococcus SB P-replete (exponential phase)	1	0.009 ± 0.001	0.0036 ± 0.0005	0.0052 ± 0.0007
	2	0.0057 ± 0.0005	0.0024 ± 0.0002	0.0033 ± 0.0003
	mean	0.007 ± 0.001	0.0030 ± 0.0004	0.0042 ± 0.0007
Prochlorococcus SB P-starved (stationary phase)	1	0.008 ± 0.001	0.0059 ± 0.0009	0.0025 ± 0.0004
	2	0.0070 ± 0.0002	0.0050 ± 0.0002	0.00203 ± 0.00007
	mean	0.0076 ± 0.0005	0.0054 ± 0.0003	0.0047 ± 0.0002

The proportions of phosphonate (Phn) and phosphate (Ph) in the cells and the P/C ratio are used to calculate the Phn-P/C and Ph-P/C ratios. Errors associated the P/C ratio are calculated using the errors associated with the %C and %P in the cells and as: $\Delta\frac{P}{C} = \frac{P}{C} \times \sqrt{(\frac{\Delta\%C}{\%C})^2 + (\frac{\Delta\%P}{\%P})^2}$. Errors associated with Phn-P/C and Ph-P/C ratios are calculated as: $\Delta\frac{Phn-P}{C} = \frac{Phn-P}{C} \times \sqrt{(\frac{\Delta P/C}{P/C})^2}$. Errors associated with the mean values are calculated using the standard error of the mean.

Table S5: Functional enrichment of polysaccharide biosynthesis genes within phosphonate biosynthesis gene clusters.

Genome	Kegg Pathway	Gene Ratio	Background Ratio	p.adjust	qvalue	KO	Gene function Description
SB	map00520	6/16	24/754	7×10-5	6×10-5	K02377, K02377	Nad-dependent epimerase dehydratase
SB	map00520	6/16	24/754	7×10-5	6×10-5	K01784	udp-glucose 4-epimerase
SB	map00520	6/16	24/754	7×10-5	6×10-5	K01710, K08679	Nucleotide sugar epimerase
SB	map00520	6/16	24/754	7×10-5	6×10-5	K01711	Gdp-mannose 4,6-dehydratase
SB	map00520	6/16	24/754	7×10-5	6×10-5	K00971, K16011	Mannose-1-phosphate guanylyltransferase
SB	map00520	6/16	24/754	7×10-5	6×10-5	K00012	Udp-glucose 6-dehydrogenase
RS40	map00520	6/28	29/755	1×10-2	1×10-2	K01784	epimerase dehydratase
RS40	map00520	6/28	29/755	1×10-2	1×10-2	K13010	DegT/DnrJ/EryC1/StrS aminotransferase family
RS40	map00520	6/28	29/755	1×10-2	1×10-2	K01654, K18430	synthase
RS40	map00520	6/28	29/755	1×10-2	1×10-2	K01791, K18429	UDP-N-acetylglucosamine 2-epimerase
RS40	map00520	6/28	29/755	1×10-2	1×10-2	K00966, K16881	Glucose-1-phosphate cytidylyltransferase
RS40	map00520	6/28	29/755	1×10-2	1×10-2	K01654, K18430	synthase

Subset of results from a Universal Enrichment Analysis implemented with the bioconductor package clusterprofiler. The remainder of the results for *Prochlorococcus* and SAR11 are available from https://github.com/slhogle/phosphonates. SB, *Prochlorococcus* sp. SB; RS40, *Candidatus Pelagibacter* sp. RS40; map00520, Amino sugar and nucleotide sugar metabolism; KO, Kegg Orthology identifier; p.adjust, Bounded False Discovery Rate (FDR) adjusted probability for hypergeometric test statistic under the null hypothesis; qvalue, direct estimate of the FDR associated with p.adjust; Gene Ratio, k/n where n is the total number of genes within 10000 nucleotides upstream/downstream of the *pepM* gene and k is the number of genes in that region annotated to the

Kegg Pathway; Background ratio, M/N where N is the total number of genes with a Kegg Orthology assignment and M is the number of Kegg Orthology assigned genes annotated to the Kegg Pathway.

Supplementary Figures

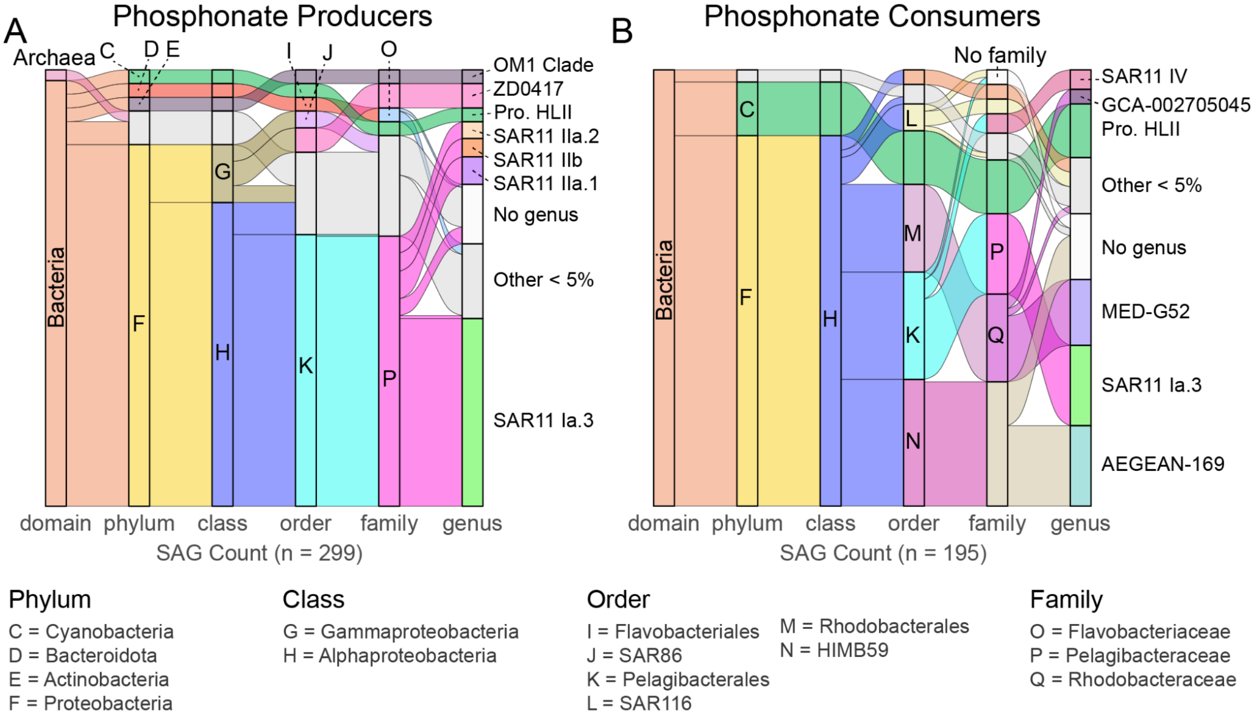

Figure S1: Genomic potential for phosphonate production and consumption in single-cell genomes from the tropical ocean
Flow diagrams representing taxonomic hierarchy of A) phosphonate producers and B) consumers from the GORG-Tropics database. Flows of the same color spanning multiple taxonomic ranks indicate that the higher ranks are entirely subsumed by the lowest rank. For example, all cyanobacterial genomes were from the *Prochlorococcus* genus. Otherwise each partition at each taxonomic level is colored uniquely. For the quantitative comparison of GORG-Tropics sample BATS248 was randomly subsampled to the median depth of the other 27 samples. Letters at each taxonomic rank refer to key marine archaea/bacterial groups (key left) and bar size is proportional to relative abundance.

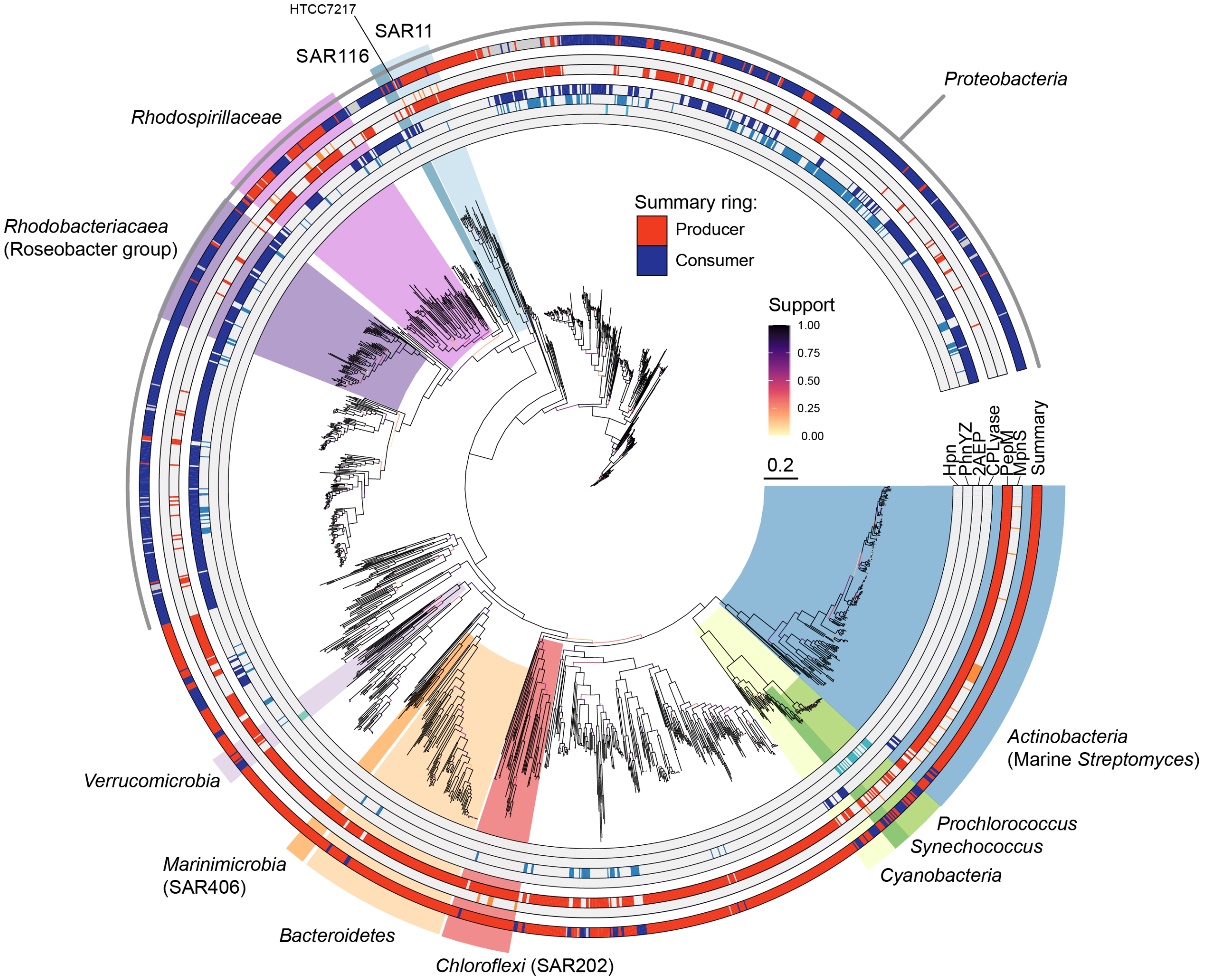

Figure S2: Distribution of phosphonate biosynthesis and utilization pathways in bacterial genomes.

Phylogeny is constructed from 120 concatenated, single-copy marker genes from 1890 bacterial genomes from MARMICRODB containing either a phosphonate catabolism or phosphonate biosynthesis pathway. Scale bar is 0.2 amino acid substitution and the tree is unrooted. Monophyletic taxonomic groups with marine representatives are highlighted. The presence of four different phosphonate catabolic pathways (Hpn: Phosphonate catabolism via HpnWXZ, PhnYZ: phosphite or methylphosphonate catabolism via PhnYZ (65), 2AEP: 2-aminoethylphosphonate catabolism via phosphonoacetate, CPLyase: multisubunit C-P lyase system) is displayed in blue on an inner ring while phosphonate/methylphosphonate biosynthesis pathways (*pepM* and *mpnS*) are shown in red on the middle ring. The outer summary ring indicates whether a genome contains at least one catabolic pathway (blue), a phosphonate biosynthesis pathway (red), or both (dark grey, < 0.5% of all genomes). For simplification, the MpnS category also includes the functionally related enzyme HepDI (63). For reference, HTCC7217 from the SAR11 clade 1a3 is both a producer and consumer.

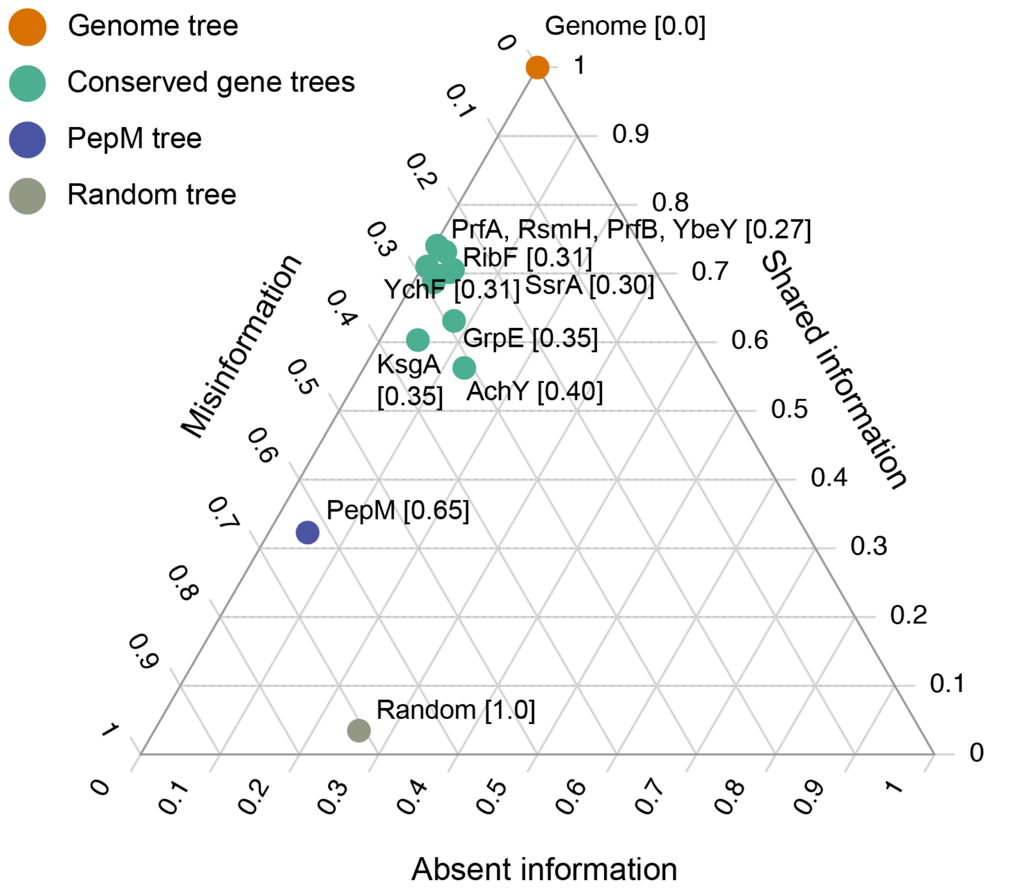

Figure S3: Similarities between genome, conserved gene families, *pepM*, and random tree topologies.

Ternary plot displaying similarities between different phylogenies using Mutual Clustering Information (MCI). Phylogenies more similar to the multi-gene concatenated genome phylogeny are positioned vertically towards the 100% shared information vertex. The 'Absent information' axis represents the resolved MCI relative to the theoretical maximum MCI. The Misinformation axis shows the amount of incorrect MCI between trees. The blue point is the comparison between the *pepM* phylogeny and the genome phylogeny, and the grey point is a comparison between a completely random phylogeny and the genome phylogeny. The green points are comparisons between conserved gene families (not included in the genome phylogeny) and the genome phylogeny. Each gene tree vs. genome tree comparison is presented alongside the MCI expressed as a distance (in brackets), but rescaled such that the expected distance between two completely random trees equals 1.

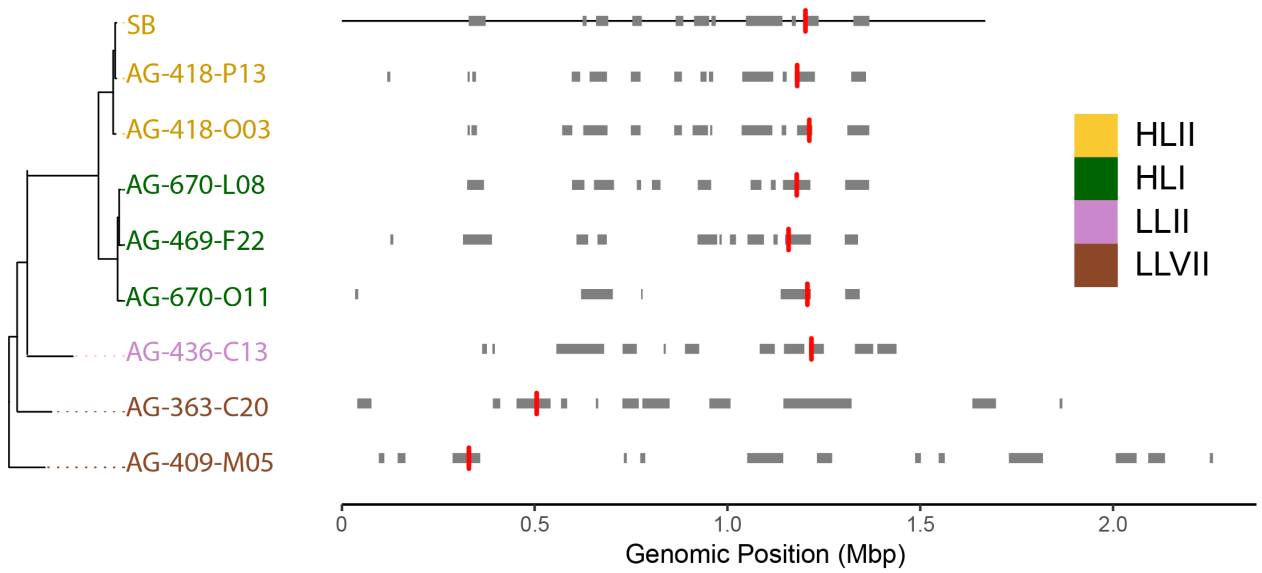

Figure S4: Distribution of phosphonate biosynthesis gene clusters within *Prochlorococcus* genomic islands

Includes a subset of all *Prochlorococcus* genomes with i) high enough completeness and low enough contig fragmentation for island prediction (see methods) and ii) that contain phosphonate biosynthesis genes. Genomes are ordered by phylogeny (left) and colored by ecotype/clade (right). Grey bars denote the location of predicted genomic islands and red vertical lines denote the location of phosphonate biosynthesis clusters within predicted genomic islands. *Prochlorococcus* SB genome assembly length is shown as a black horizontal line. The assembly lengths of single cell genomes are omitted due to incompleteness and contig fragmentation. Single cell genome contigs and genomic islands are arbitrarily ordered to *Prochlorococcus* SB.

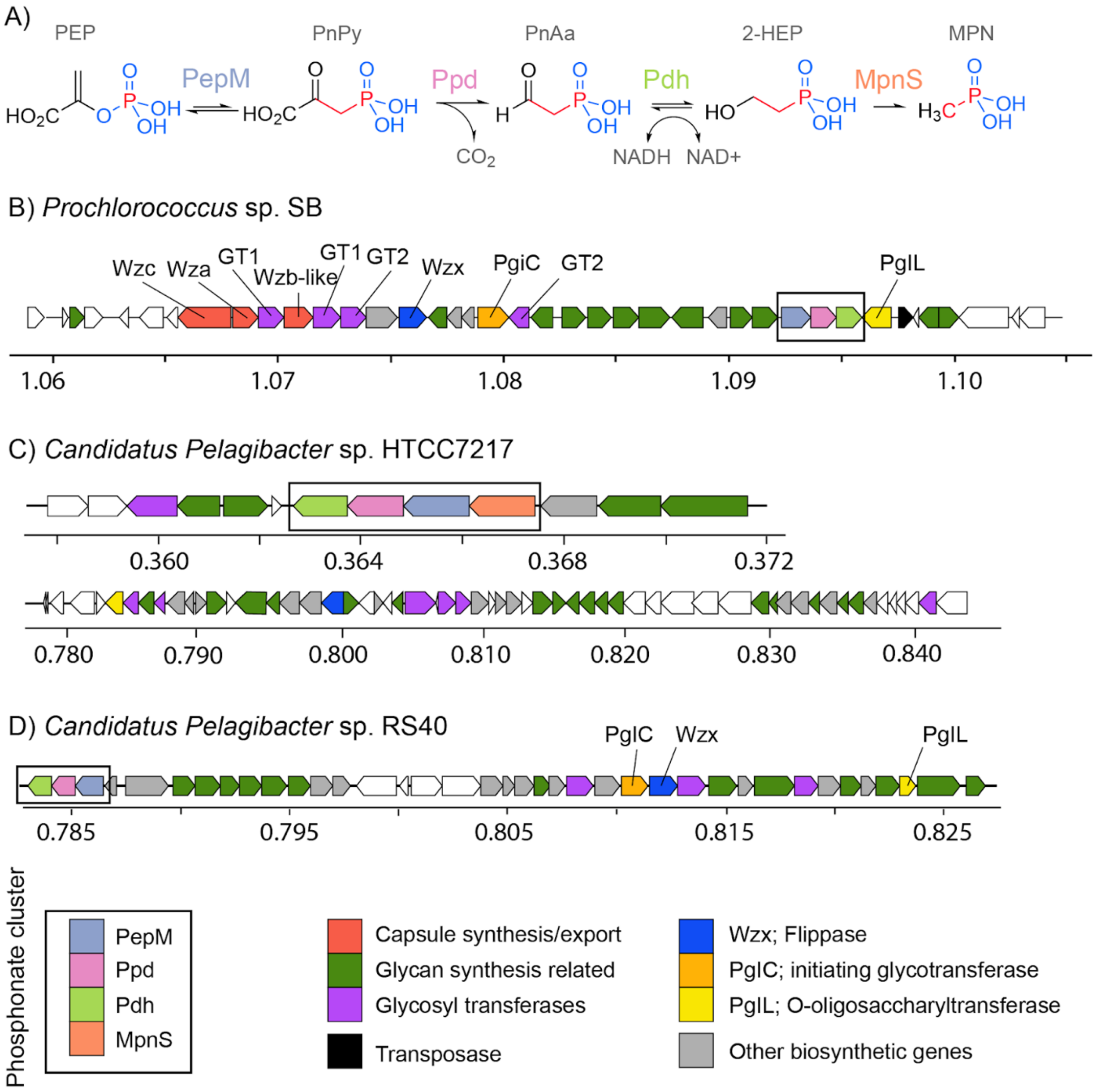

Figure S5: Polysaccharide and phosphonate biosynthesis in *Prochlorococcus* and SAR11 isolate genomes

A) same reaction diagram as in Figure 1 with chemical intermediates (black) and catalyzing enzymes (color). **B-D)** Putative phosphonoglycoprotein biosynthesis gene clusters in *Prochlorococcus* SB, *Candidatus Pelagibacter* sp. HTCC7217, and *Candidatus Pelagibacter* sp. RS40 genomes annotated by antiSMASH (105) and eggNOG mapper (106). Genome coordinates are $\times 10^6$ base pairs. Phosphonate biosynthetic genes are colored by enzyme as in **A)** and are shown in black boxes. Genes involved in modular biosynthesis of sugars are shown in red, green, and purple. PgiC is the putative initiating glycotransferase, which links the first glycan subunit to a lipid carrier, Wzx is a flippase which translocates the assembled glycan-bound lipid carrier to the periplasmic face, and PglL is an O-oligosaccharyltransferase which is the critical enzyme moving the assembled glycan chain to the final acceptor protein. Other biosynthetic genes (grey) have functional predictions not directly related to glycan biosynthesis.

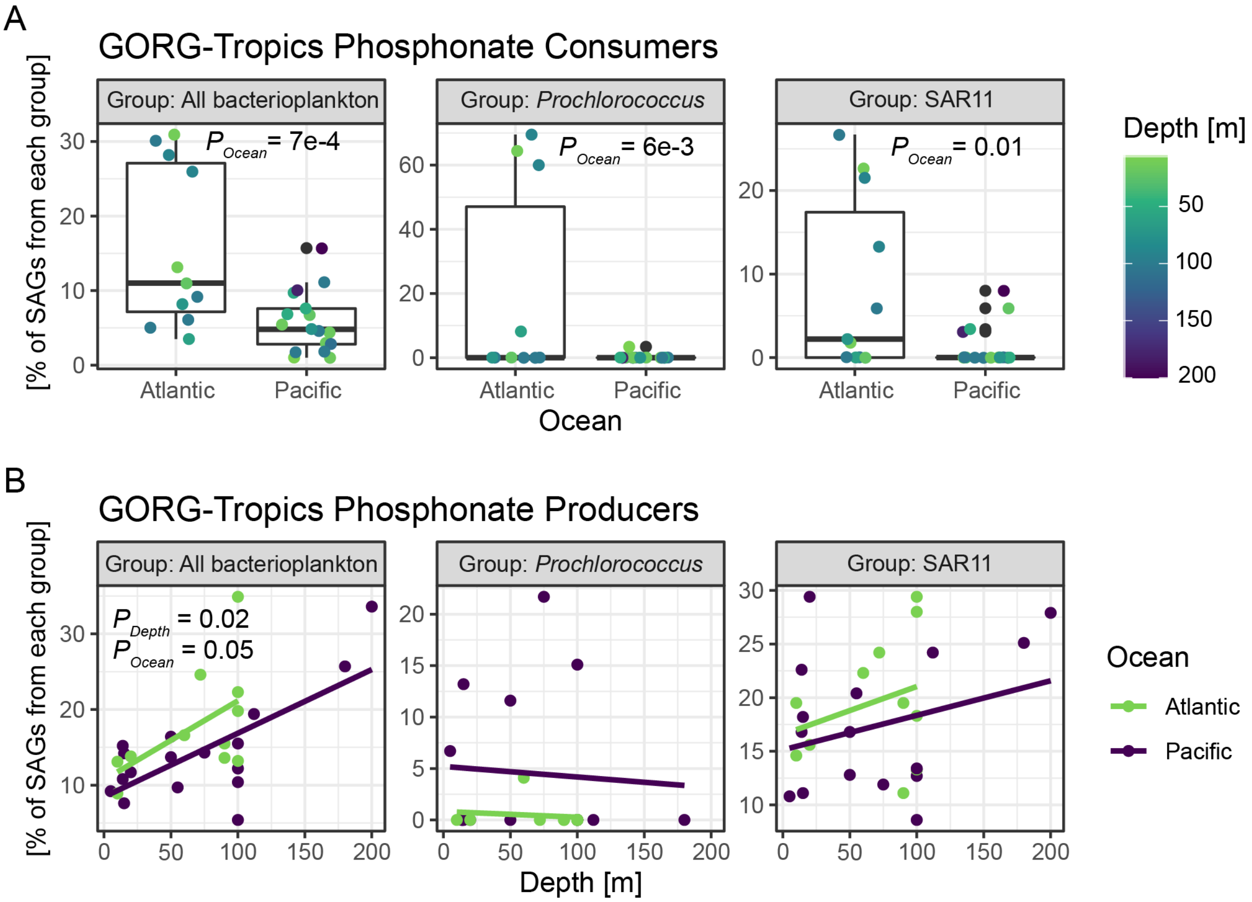

Figure S6: Phosphonate consumers and producers in 28 GORG-Tropics samples

Same as in Figure 3A, B but analyzing *Prochlorococcus* and SAR11 separately. Proportions [%] are total producers or consumers divided by the total number GORG assemblies and are corrected using the estimated sequence recovery from assemblies (see methods). Each dot represents a single GORG sample location. P values for covariates in beta-binomial regression are displayed if statistically significant ($P \le 0.05$). Beta-binomial regression all consumers; Ocean - Est=-1.04, Err=0.27, t=-3.81, P=7e-4; link=logit; log L = -80.425, df=4, resid df=24. Beta-binomial regression *Prochlorococcus* consumers; Ocean - Est=-3.50, Err=1.15, t=-3.04, P=6e-3; link=logit; log L = -20.181, df=4, resid df=21. Beta-binomial regression SAR11 consumers; Ocean - Est=-1.73, Err=0.65, t=-2.66, P=0.01; link=logit; log L = -41.916, df=4, resid df=24. Beta-binomial regression all producers; Depth - Est=0.0036, Err=0.0014, t=2.604, P=0.02; Ocean - Est=-0.30, Err=0.14, t=-2.08, P=0.05; link=logit; log L=-78.179, df=4, resid df=24.

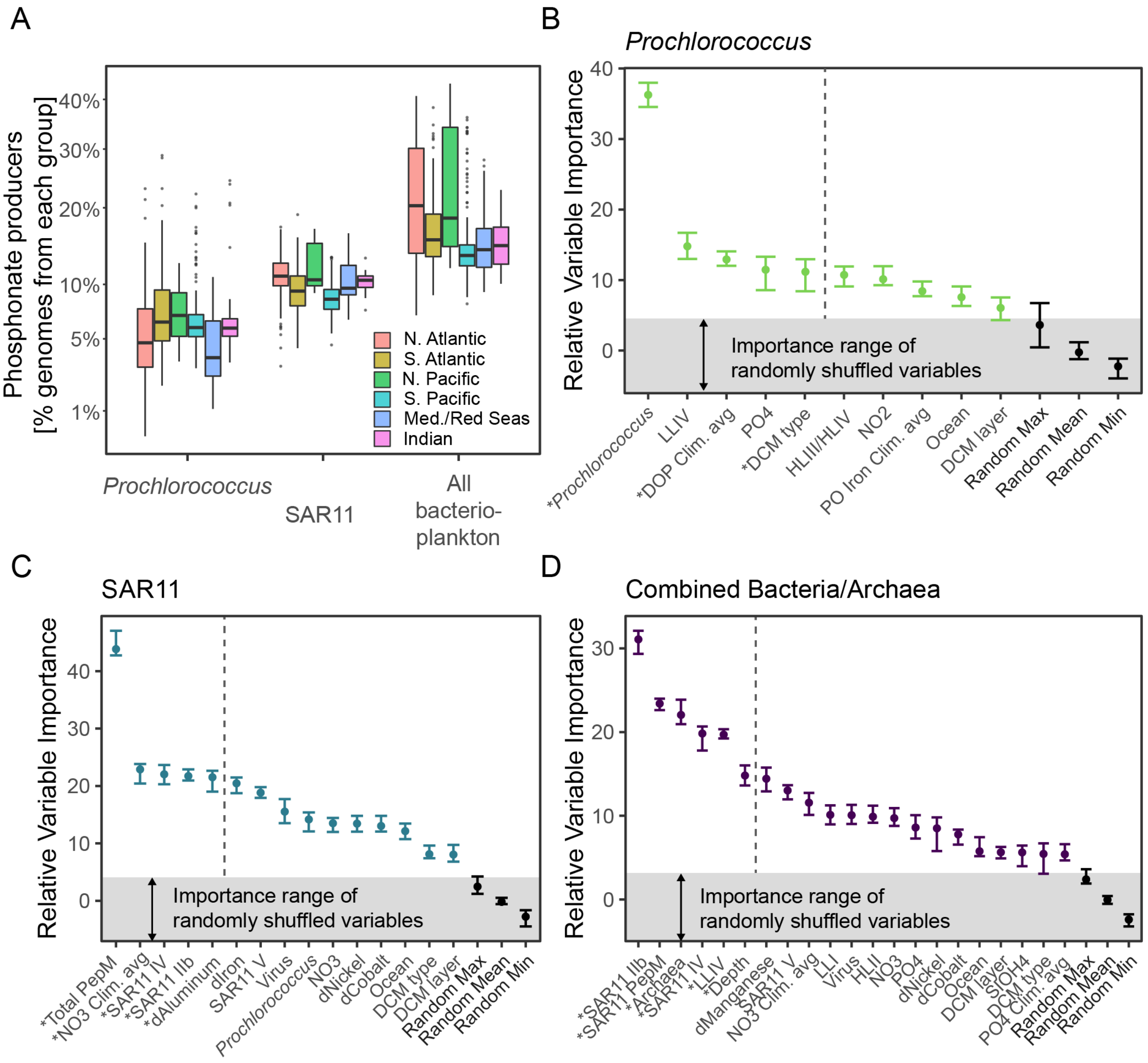

Figure S7: Environmental factors driving the relative abundance of *pepM* in *Prochlorococcus*, SAR11, and combined Bacteria/Archaea

A) Same as in Figure 3A but binned by ocean region. There is no statistically significant difference between ocean regions for *Prochlorococcus*, SAR11, or combined Bacteria/Archaea *pepM* relative abundance. Black line is the median value, the box shows the 25th and 75th percentiles, and the whiskers extend to 1.5 of the interquartile range for n metagenome samples in each group. Individual points are outliers, and the vertical axis is square-root transformed. **B-D)** Random forest variable importance measures for each taxonomic group. Point ranges show median and maximum/minimum for 100 random permutations of the variable importance algorithm. Only informative variables for predicting *pepM* distributions are displayed. Randomized controls in black represent the distribution of the median, minimum, and maximum importance of randomly shuffled variables at each algorithm iteration and can be interpreted as the baseline variable importance expected simply due to chance. Asterisks indicate whether a variable was also statistically significant in a parametric beta-binomial regression (Table S1). The top five predictive variables are demarcated by a dashed line. *Prochlorococcus*, Archaea, and viruses are units percentage of all successfully mapped reads to MARMICRODB. Clades HLII, LLI, LLIV, HLIII/HLIV, SAR11 IV, SAR11 IIb, SAR11 V, are percentage of reads mapped to *Prochlorococcus* and SAR11, respectively. Clim. avg, climatological average from the MIT Darwin model; DCM depth range, deep chlorophyll maximum layer depth category – 1=shallow, 6=deep. *pepM* units are the percentage of *Prochlorococcus*, SAR11, or all genomes with the trait.

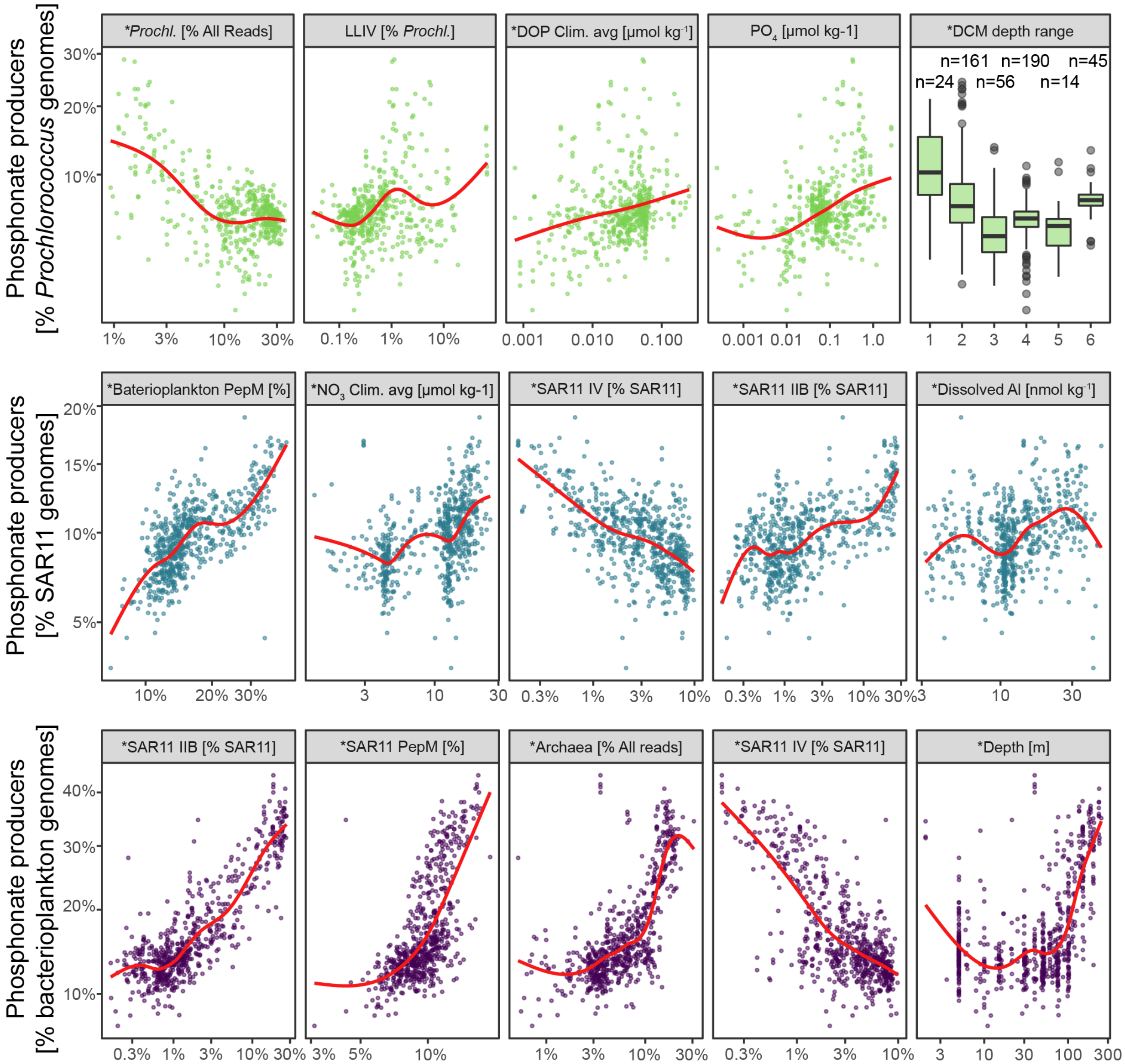

Figure S8: Relationship between *Prochlorococcus*, SAR11, and combined Bacteria/Archaea *pepM* relative abundance and the top five biotic/abiotic covariates for each taxonomic group

Phosphonate producer relative abundance (vertical axis) vs environmental features (horizontal axis) for the top five most informative variables from random forest regressions of each taxonomic group (Figure 3). Each point is a metagenomic observation. Climatological averages are from the MIT Darwin model while the other biotic/abiotic variables are in situ measurements from GEOTRACES and Tara Oceans. Red lines are local polynomial regression fits to the data. Asterisks indicate whether a variable was also statistically significant in a parametric beta-binomial regression (Table S1). *Prochlorococcus* and Archaea are percentage of all successfully mapped reads to MARMICRODB. Clades LLIV, SAR11 IV, SAR11 IIb are percentage of reads mapped to *Prochlorococcus* and SAR11, respectively. Clim. avg, climatological average from the MIT Darwin model; DCM depth range, deep chlorophyll maximum layer depth category – 1=shallow, 6=deep. *pepM* units are the percentage of *Prochlorococcus*, SAR11, or all genomes with the trait.

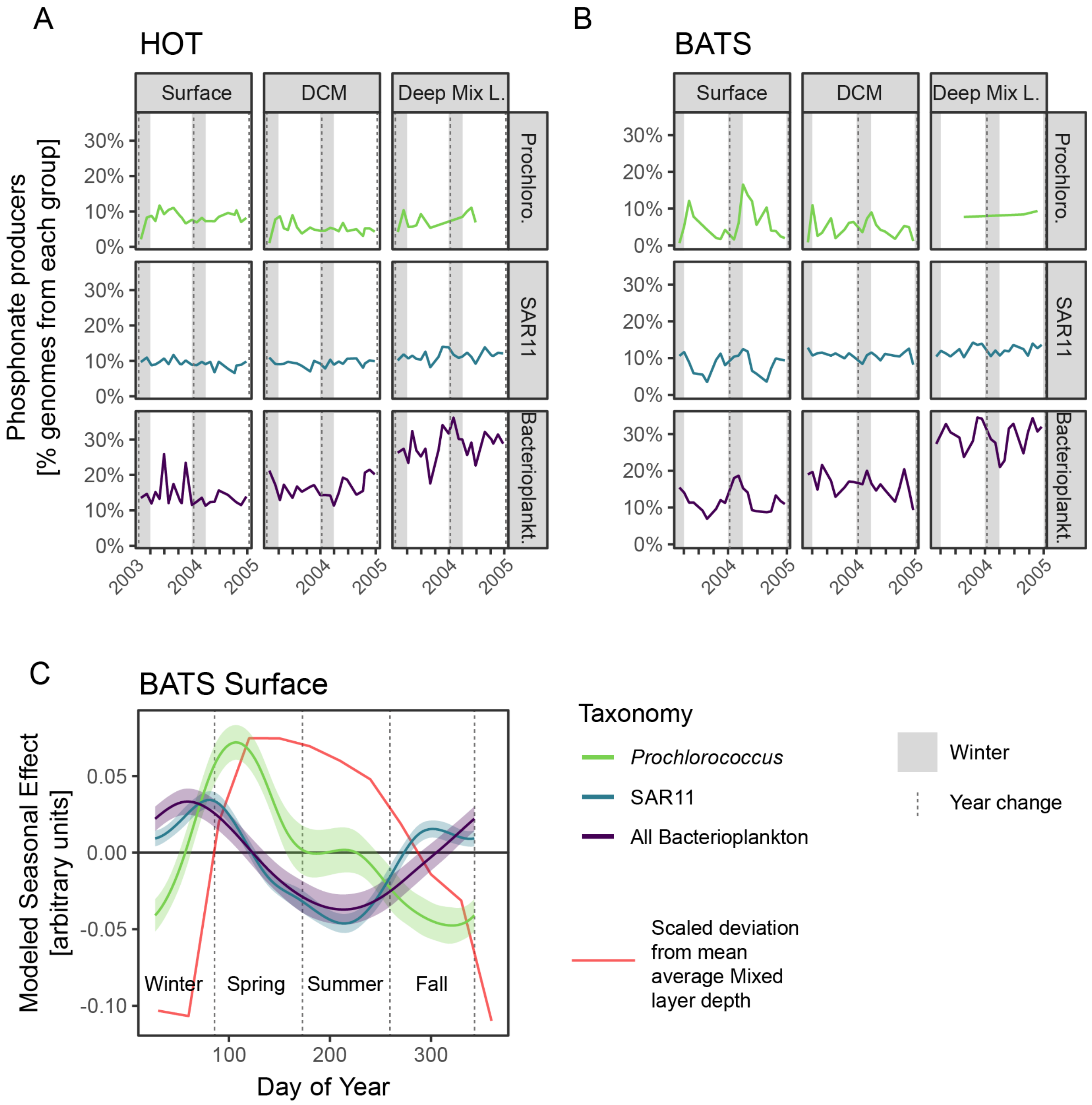

Figure S9: Time series of *pepM* relative abundance at the Hawaii Ocean Time-series (HOT) and the Bermuda Atlantic Time Series (BATS)

pepM relative abundance shown by depth range (horizontal grid axis, DCM = deep chlorophyll maximum layer, Deep Mix L. = deep mixed layer, 1% photosynthetically active radiation) and by taxonomic grouping (vertical grid) at **A)** HOT and **B)** BATS. Tick marks on the horizontal axis mark seasonal boundaries as in subplot C, grey bars denote the winter season, and dashed vertical lines mark year boundaries. **C)** The modeled seasonal effect at BATS surface on the relative abundance of *pepM* of each taxonomic grouping. [GAM SAR11, family=Gamma, link=identity: s(dayOfYear), edf=5.4, Ref. df=8, F=9.8, P=7e-5. GAM *Prochlorococcus*, family=Gamma, link=identity: s(dayOfYearDCM), edf=3.8, Ref. df=8, F=4.5, P=0.001, GAM Bacterioplankton, family=Gamma, link=identity: s(dayOfYearDCM), edf=3.2, Ref. df=8, F=5.0, P=2e-4]. The red line shows the scaled deviation from the annual mean of the mixed layer depth at BATS for the time range in **B)**. For example, a negative deviation indicates that the mixed layer depth is deeper than the annual mean.

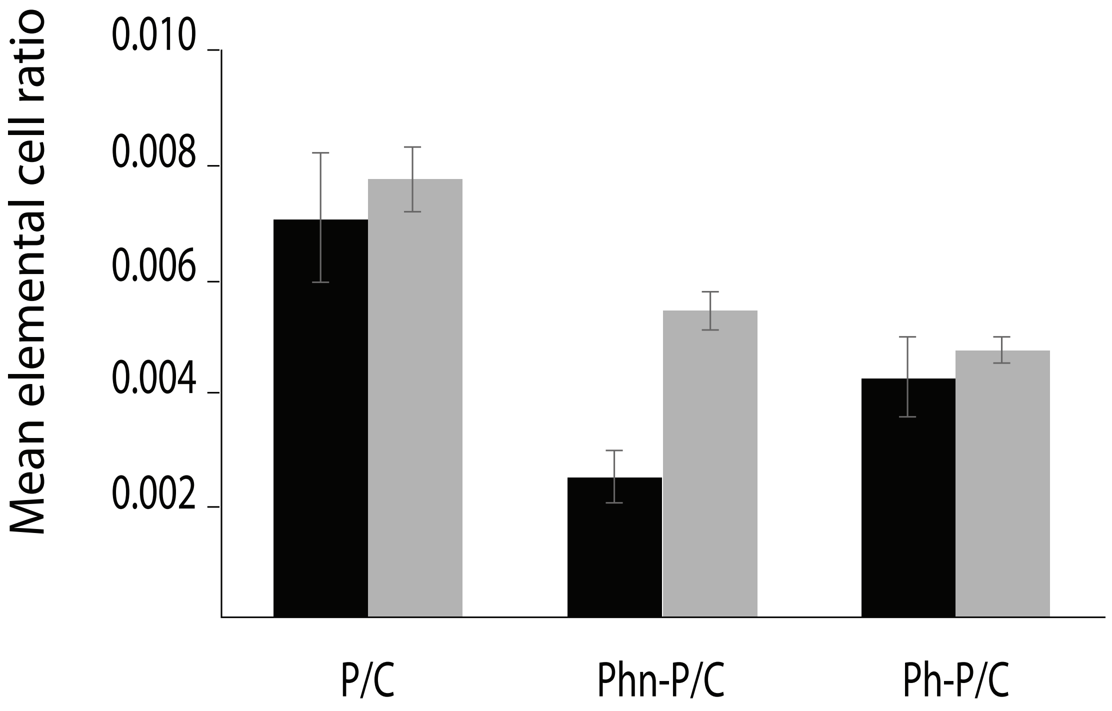

Figure S10: Variations of *Prochlorococcus* SB P/C, Phn-P/C and Ph-P/C ratios between in exponential (P-replete; black) and stationary phase (P-starved; grey)

When *Prochlorococcus* SB cells are P-starved, their P/C and Ph-P/C ratios are not measurably different from cells grown under P-replete conditions (left, right), whereas their Phn-P/C ratio (center) increases, indicating an increase in phosphonate content (Table S4). As before, errors associated with the mean values are calculated using the standard error of the mean.

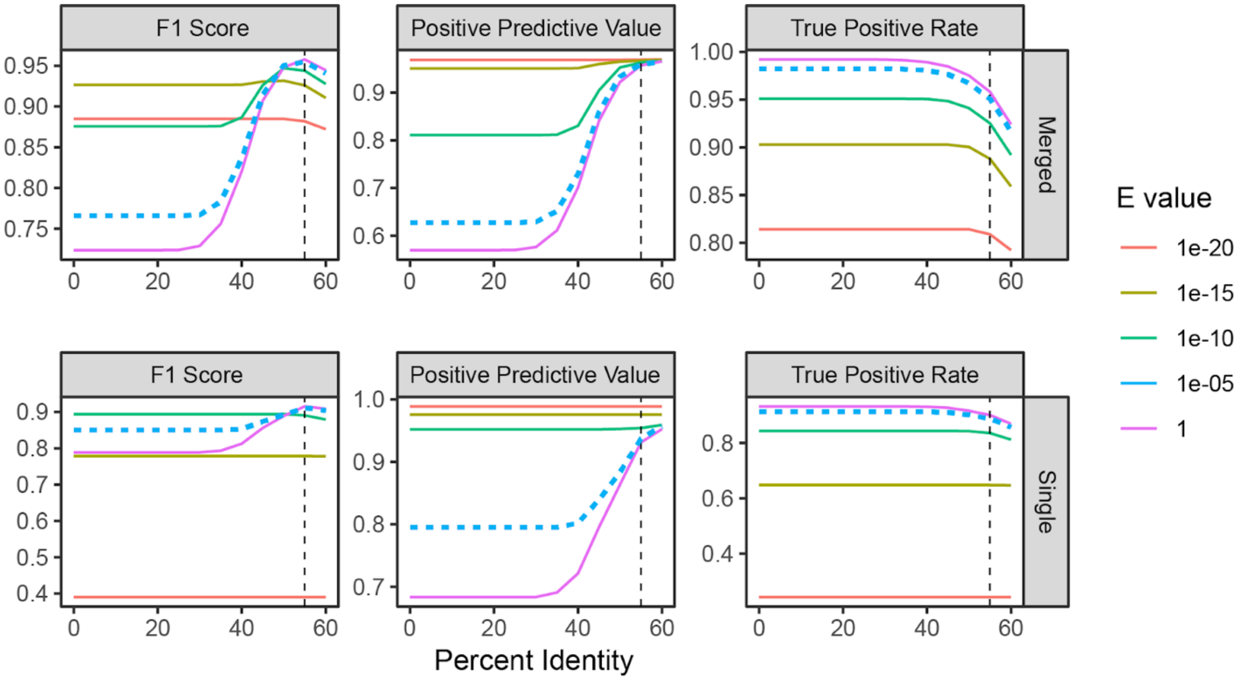

Figure S11: Empirical determination of similarity cutoffs for identifying *pepM*-derived metagenomic short sequencing reads.

F1 score, Positive Predictive Value, and True Positive Rate for classifying *pepM* from mock metagenomes simulated from 150 bp Illumina paired-end reads. Results are shown both for merged read pairs (top), and the best scoring read from an unmerged read pair (bottom). The True Postive Rate measures the proportion of “true” simulated *pepM* reads correctly identified by a E value/Percent Identity cutoff combination while Positive Predictive Value measures the proportion of “true” simulated *pepM* reads that are correctly identified out of all reads passing the E value/Percent Identity cutoff combination. The F1 score is the harmonic mean of precision and sensitivity. Cutoffs were empirically chosen to maximize the F1 score resulting in an E value threshold of 1e-5 (dashed blue line) and a Percent Identity cutoff of 55% (dashed vertical line).

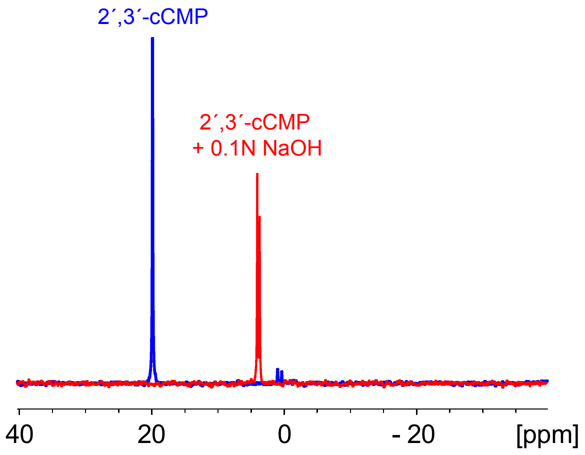

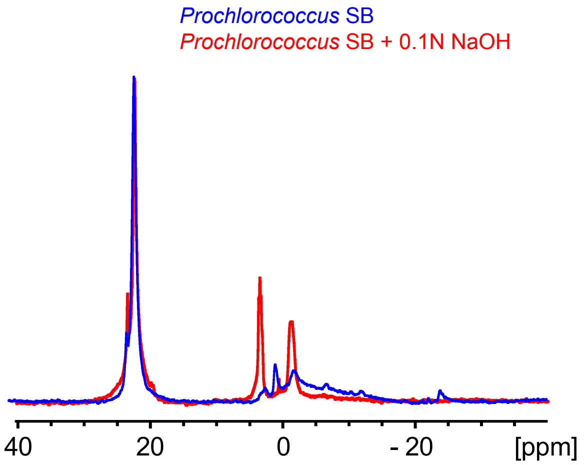

Figure S12: Base-catalzyed degradation of 2´,3´-cyclic nucleotide monophosphates. (Top) The ^{31}P NMR spectrum of 2´,3´-cyclic cystidine monophosphate (2´,3´-cCMP; blue trace) displays a sharp peak at 20.0 ppm. Treatment with 0.1N NaOH for 2 hr degrades 2´,3´-cCMP to a mixture of 2´- and 3´- (acyclic)CMP which appears as two nearly equal sized peaks at ~ 4 ppm (red trace). (Bottom) Parallel treatment *of Prochlorococcus* SB cells (blue trace) with 0.1N NaOH sharpens the phosphate esters region of the spectrum (red trace, -25 to 5 ppm), but does not change the ratio of the phosphonate (18-27 ppm) to phosphate ester (-25 to 5 ppm) integrals (61/38 before addition of base, 61/38 after). The *Prochlorococcus* SB sample used for this experiment was grown under nutrient replete conditions and harvested in stationary phase.

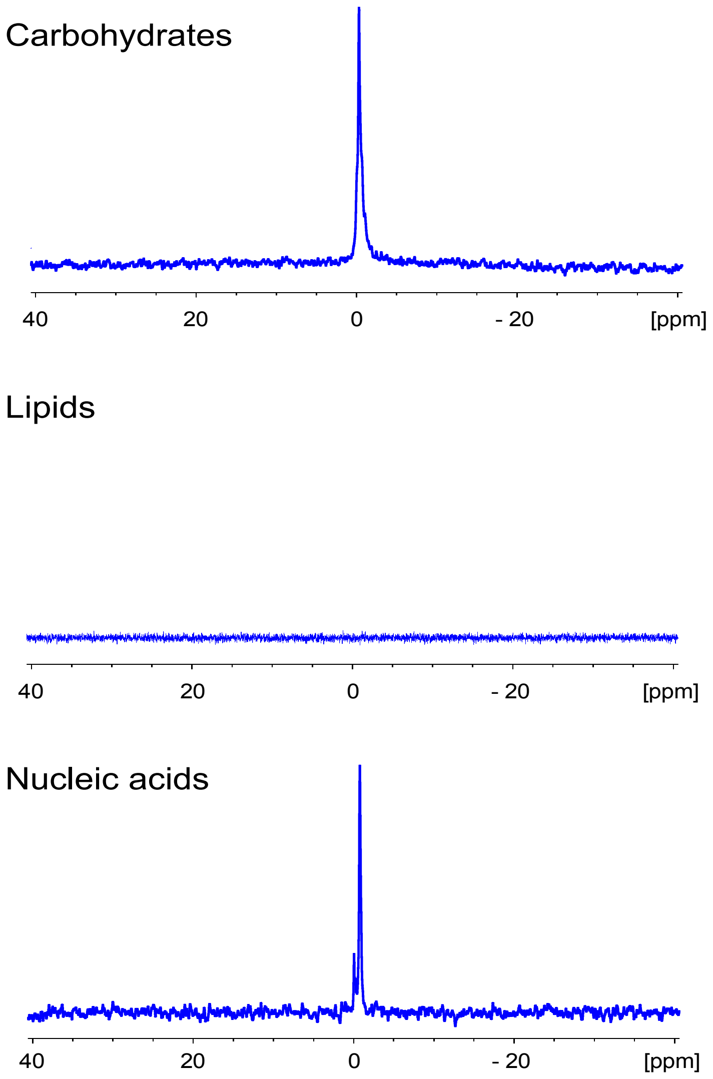

Figure S13: ^{31}P NMR spectra of major biochemical fractions extracted from *Prochlorococcus* SB. (Top) Carbohydrates extracted with cold aqueous trichloroacetic acid include phosphoglycans that appear at ~ 0 ppm, while no phosphorus was detected in the lipid fraction extracted with aqueous ethanol (Center). As expected, phosphate esters were recovered in the nucelic acid fraction (~ 0 ppm, Bottom). Phosphonate and some phosphate esters appear in the protien fraction (Fig. 4).

Supplementary References

1. L. Alonso-Sáez, J. M. Gasol, Seasonal variations in the contributions of different bacterial groups to the uptake of low-molecular-weight compounds in northwestern Mediterranean coastal waters. *Appl. Environ. Microbiol.* **73**, 3528–3535 (2007).

2. J. W. Becker, S. L. Hogle, K. Rosendo, S. W. Chisholm, Co-culture and biogeography of Prochlorococcus and SAR11. *ISME J.* **13**, 1506–1519 (2019).

3. P. J. Keeling, *et al.*, The Marine Microbial Eukaryote Transcriptome Sequencing Project (MMETSP): illuminating the functional diversity of eukaryotic life in the oceans through transcriptome sequencing. *PLoS Biol.* **12**, e1001889 (2014).

4. W. W. Metcalf, *et al.*, Synthesis of methylphosphonic acid by marine microbes: a source for methane in the aerobic ocean. *Science* **337**, 1104–1107 (2012).

5. S. Neuer, *et al.*, Differences in the biological carbon pump at three subtropical ocean sites. *Geophys. Res. Lett.* **29**, 32-1-32–4 (2002).

6. S. J. Giovannoni, K. L. Vergin, Seasonality in Ocean Microbial Communities. *Science* **335**, 671–676 (2012).

7. D. M. Karl, M. J. Church, Microbial oceanography and the Hawaii Ocean Time-series programme. *Nat. Rev. Microbiol.* **12**, 699–713 (2014).

8. J. Wu, W. Sunda, E. A. Boyle, D. M. Karl, Phosphate depletion in the western North Atlantic Ocean. *Science* **289**, 759–762 (2000).

9. E. A. Boyle, B. A. Bergquist, R. A. Kayser, N. Mahowald, Iron, manganese, and lead at Hawaii Ocean Time-series station ALOHA: Temporal variability and an intermediate water hydrothermal plume. *Geochim. Cosmochim. Acta* **69**, 933–952 (2005).

10. P. N. Sedwick, *et al.*, Iron in the Sargasso Sea (Bermuda Atlantic Time-series Study region) during summer: Eolian imprint, spatiotemporal variability, and ecological implications. *Global Biogeochem. Cycles* **19**, GB4006 (2005).

11. M. L. Coleman, S. W. Chisholm, Ecosystem-specific selection pressures revealed through comparative population genomics. *Proc. Natl. Acad. Sci. U. S. A.* **107**, 18634–18639 (2010).

12. A. C. Martiny, M. L. Coleman, S. W. Chisholm, Phosphate acquisition genes in Prochlorococcus ecotypes: evidence for genome-wide adaptation. *Proc. Natl. Acad. Sci. U. S. A.* **103**, 12552–12557 (2006).

13. K. L. Vergin, *et al.*, High-resolution SAR11 ecotype dynamics at the Bermuda Atlantic Time-series Study site by phylogenetic placement of pyrosequences. *ISME J.* **7**, 1322–1332 (2013).

14. K. J. Orians, K. W. Bruland, The biogeochemistry of aluminum in the Pacific Ocean. *Earth Planet. Sci. Lett.* **78**, 397–410 (1986).

15. M. B. Karner, E. F. DeLong, D. M. Karl, Archaeal dominance in the mesopelagic zone of

the Pacific Ocean. *Nature* **409**, 507–510 (2001).

16. C. E. Nelson, C. A. Carlson, C. S. Ewart, E. R. Halewood, Community differentiation and population enrichment of Sargasso Sea bacterioplankton in the euphotic zone of a mesoscale mode-water eddy. *Environ. Microbiol.* **16**, 871–887 (2014).

17. M. van Hulten, *et al.*, Manganese in the west Atlantic Ocean in the context of the first global ocean circulation model of manganese. *Biogeosciences* **14**, 1123–1152 (2017).

18. L. C. Kolowith, E. D. Ingall, R. Benner, Composition and cycling of marine organic phosphorus. *Limnol. Oceanogr.* **46**, 309–320 (2001).

19. R. Benner, "Chemical Composition and Reactivity" in *Biogeochemistry of Marine Dissolved Organic Matter*, (Elsevier, 2002), pp. 59–90.

20. D. M. Karl, K. M. Björkman, "Chapter 5 - Dynamics of Dissolved Organic Phosphorus" in *Biogeochemistry of Marine Dissolved Organic Matter (Second Edition)*, D. A. Hansell, C. A. Carlson, Eds. (Academic Press, 2015), pp. 233–334.

21. D. J. Repeta, *et al.*, Marine methane paradox explained by bacterial degradation of dissolved organic matter. *Nat. Geosci.* **9**, 884–887 (2016).

22. B. A. S. Van Mooy, *et al.*, Phosphorus cycling. Major role of planktonic phosphate reduction in the marine phosphorus redox cycle. *Science* **348**, 783–785 (2015).

23. A. E. Santoro, R. A. Richter, C. L. Dupont, Planktonic Marine Archaea. *Ann. Rev. Mar. Sci.* **11**, 131–158 (2019).

24. A. E. Santoro, *et al.*, Thaumarchaeal ecotype distributions across the equatorial Pacific Ocean and their potential roles in nitrification and sinking flux attenuation. *Limnol. Oceanogr.* **62**, 1984–2003 (2017).

25. A. E. Santoro, *et al.*, Nitrification and nitrous oxide production in the offshore waters of the Eastern tropical south pacific. *Global Biogeochem. Cycles* **35** (2021).

26. K. L. Casciotti, A. E. Santoro, Water column amoA and Nitrospina-like 16S rRNA gene abundances from qPCR in the Eastern Tropical South Pacific using seawater collected on R/V Atlantis (AT15-61) cruise in Jan-Feb 2010 and R/V Melville (MV1104) cruise in Mar-Apr 2011 (2021) https:/doi.org/10.26008/1912/BCO-DMO.826781.1.

27. J. J. Polovina, E. A. Howell, M. Abecassis, Ocean's least productive waters are expanding. *Geophys. Res. Lett.* **35**, 270 (2008).

28. T. B. Meador, *et al.*, Carbon recycling efficiency and phosphate turnover by marine nitrifying archaea. *Sci Adv* **6**, eaba1799 (2020).

29. D. L. Kirchman, H. Elifantz, A. I. Dittel, R. R. Malmstrom, M. T. Cottrell, Standing stocks and activity of Archaea and Bacteria in the western Arctic Ocean. *Limnol. Oceanogr.* **52**, 495–507 (2007).

30. S. T. Dyhrman, C. R. Benitez-Nelson, E. D. Orchard, S. T. Haley, P. J. Pellechia, A microbial source of phosphonates in oligotrophic marine systems. *Nat. Geosci.* **2**, 696–699 (2009).

31. C. S. Davis, D. J. McGillicuddy Jr, Transatlantic abundance of the N2-fixing colonial cyanobacterium Trichodesmium. *Science* **312**, 1517–1520 (2006).

32. E. Breitbarth, A. Oschlies, J. LaRoche, Physiological constraints on the global distribution of *Trichodesmium* – effect of temperature on diazotrophy. *Biogeosciences* **4**, 53–61 (2007).

33. D. G. Capone, J. P. Zehr, H. W. Paerl, B. Bergman, E. J. Carpenter, Trichodesmium, a Globally Significant Marine Cyanobacterium. *Science* **276**, 1221–1229 (1997).

34. S. A. Sañudo-Wilhelmy, *et al.*, Phosphorus limitation of nitrogen fixation by Trichodesmium in the central Atlantic Ocean. *Nature* **411**, 66–69 (2001).

35. S. J. Biller, P. M. Berube, D. Lindell, S. W. Chisholm, Prochlorococcus: The structure and function of collective diversity. *Nat. Rev. Microbiol.* **13**, 13–27 (2015).

36. M. Heldal, D. J. Scanlan, S. Norland, F. Thingstad, N. H. Mann, Elemental composition of single cells of various strains of marine Prochlorococcus and Synechococcus using X-ray microanalysis. *Limnol. Oceanogr.* **48**, 1732–1743 (2003).

37. P. Flombaum, Present and future global distributions of the marine Cyanobacteria Prochlorococcus and Synechococcus. *Proc. Natl. Acad. Sci. U. S. A.* **110**, 9824–9829 (2013).

38. C. Grob, *et al.*, Elemental composition of natural populations of key microbial groups in Atlantic waters. *Environ. Microbiol.* **15**, 3054–3064 (2013).

39. M. V. Zubkov, Faster growth of the major prokaryotic versus eukaryotic CO2 fixers in the oligotrophic ocean. *Nat. Commun.* **5**, 3776 (2014).

40. R. M. Morris, *et al.*, SAR11 clade dominates ocean surface bacterioplankton communities. *Nature* **420**, 806–810 (2002).

41. S. J. Giovannoni, SAR11 Bacteria: The Most Abundant Plankton in the Oceans. *Ann. Rev. Mar. Sci.* **9**, 231–255 (2017).

42. A. E. White, S. J. Giovannoni, Y. Zhao, K. Vergin, C. A. Carlson, Elemental content and stoichiometry of SAR11 chemoheterotrophic marine bacteria. *Limnol. Oceanogr. Lett.* **4**, 44–51 (2019).

43. K. Gundersen, M. Heldal, S. Norland, D. A. Purdie, A. H. Knap, Elemental C, N, and P cell content of individual bacteria collected at the Bermuda Atlantic Time-series Study (BATS) site. *Limnol. Oceanogr.* **47**, 1525–1530 (2002).

44. D. L. Kirchman, Growth Rates of Microbes in the Oceans. *Ann. Rev. Mar. Sci.* **8**, 285–309 (2016).

45. A. Varki, *et al.*, Eds., *Essentials of Glycobiology* (Cold Spring Harbor Laboratory Press, 2016).

46. H. Nothaft, C. M. Szymanski, Protein glycosylation in bacteria: sweeter than ever. *Nat. Rev. Microbiol.* **8**, 765–778 (2010).

47. R. P. Fagan, N. F. Fairweather, Biogenesis and functions of bacterial S-layers. *Nat. Rev.*

Microbiol. **12**, 211–222 (2014).

48. I. Hug, M. F. Feldman, Analogies and homologies in lipopolysaccharide and glycoprotein biosynthesis in bacteria. *Glycobiology* **21**, 138–151 (2011).

49. S. C. Ku, B. L. Schulz, P. M. Power, M. P. Jennings, The pilin O-glycosylation pathway of pathogenic Neisseria is a general system that glycosylates AniA, an outer membrane nitrite reductase. *Biochem. Biophys. Res. Commun.* **378**, 84–89 (2009).

50. A. Vik, *et al.*, Broad spectrum O-linked protein glycosylation in the human pathogen Neisseria gonorrhoeae. *Proc. Natl. Acad. Sci. U. S. A.* **106**, 4447–4452 (2009).

51. C. M. Fletcher, M. J. Coyne, O. F. Villa, M. Chatzidaki-Livanis, L. E. Comstock, A general O-glycosylation system important to the physiology of a major human intestinal symbiont. *Cell* **137**, 321–331 (2009).

52. T. A. M. Bharat, *et al.*, Structure of the hexagonal surface layer on Caulobacter crescentus cells. *Nat Microbiol* **2**, 17059 (2017).

53. A. von Kügelgen, *et al.*, In Situ Structure of an Intact Lipopolysaccharide-Bound Bacterial Surface Layer. *Cell* **180**, 348-358.e15 (2020).

54. R. G. Lees-Miller, *et al.*, A common pathway for O-linked protein-glycosylation and synthesis of capsule in Acinetobacter baumannii. *Mol. Microbiol.* **89**, 816–830 (2013).

55. J. A. Iwashkiw, N. F. Vozza, R. L. Kinsella, M. F. Feldman, Pour some sugar on it: the expanding world of bacterial protein O-linked glycosylation. *Mol. Microbiol.* **89**, 14–28 (2013).

56. B. Hove-Jensen, D. L. Zechel, B. Jochimsen, Utilization of glyphosate as phosphate source: biochemistry and genetics of bacterial carbon-phosphorus lyase. *Microbiol. Mol. Biol. Rev.* **78**, 176–197 (2014).

57. M. G. Pachiadaki, *et al.*, Charting the Complexity of the Marine Microbiome through Single-Cell Genomics. *Cell* **179**, 1623-1635.e11 (2019).

58. D. H. Haft, *et al.*, TIGRFAMs and Genome Properties in 2013. *Nucleic Acids Res.* **41**, D387-95 (2013).

59. X. Yu, *et al.*, Diversity and abundance of phosphonate biosynthetic genes in nature. *Proc. Natl. Acad. Sci. U. S. A.* **110**, 20759–20764 (2013).

60. C. C. H. Chen, *et al.*, Structure and kinetics of phosphonopyruvate hydrolase from Variovorax sp. Pal2: new insight into the divergence of catalysis within the PEP mutase/isocitrate lyase superfamily. *Biochemistry* **45**, 11491–11504 (2006).

61. J. P. Cioni, *et al.*, Cyanohydrin phosphonate natural product from Streptomyces regensis. *J. Nat. Prod.* **77**, 243–249 (2014).

62. K.-S. Ju, *et al.*, Discovery of phosphonic acid natural products by mining the genomes of 10,000 actinomycetes. *Proc. Natl. Acad. Sci. U. S. A.* **112**, 12175–12180 (2015).

63. D. A. Born, *et al.*, Structural basis for methylphosphonate biosynthesis. *Science* **358**, 1336–

1339 (2017).

64. K. Katoh, D. M. Standley, MAFFT multiple sequence alignment software version 7: improvements in performance and usability. *Mol. Biol. Evol.* **30**, 772–780 (2013).

65. O. A. Sosa, J. R. Casey, D. M. Karl, Methylphosphonate Oxidation in Prochlorococcus Strain MIT9301 Supports Phosphate Acquisition, Formate Excretion, and Carbon Assimilation into Purines. *Appl. Environ. Microbiol.* **85** (2019).

66. A. Martinez, G. W. Tyson, E. F. Delong, Widespread known and novel phosphonate utilization pathways in marine bacteria revealed by functional screening and metagenomic analyses. *Environ. Microbiol.* **12**, 222–238 (2010).

67. W. Ding, F. Baumdicker, R. A. Neher, panX: pan-genome analysis and exploration. *Nucleic Acids Res.* (2017).

68. A. Milanese, *et al.*, Microbial abundance, activity and population genomic profiling with mOTUs2. *Nat. Commun.* **10**, 1014 (2019).

69. M. Steinegger, J. Söding, MMseqs2 enables sensitive protein sequence searching for the analysis of massive data sets. *Nat. Biotechnol.* **35**, 1026–1028 (2017).

70. S. Capella-Gutiérrez, J. M. Silla-Martínez, T. Gabaldón, trimAl: a tool for automated alignment trimming in large-scale phylogenetic analyses. *Bioinformatics* **25**, 1972–1973 (2009).

71. P.-A. Chaumeil, A. J. Mussig, P. Hugenholtz, D. H. Parks, GTDB-Tk: a toolkit to classify genomes with the Genome Taxonomy Database. *Bioinformatics* (2019) https:/doi.org/10.1093/bioinformatics/btz848.

72. D. H. Parks, *et al.*, A standardized bacterial taxonomy based on genome phylogeny substantially revises the tree of life. *Nat. Biotechnol.* (2018).

73. M. N. Price, P. S. Dehal, A. P. Arkin, FastTree 2--approximately maximum-likelihood trees for large alignments. *PLoS One* **5**, e9490 (2010).

74. S. Whelan, N. Goldman, A general empirical model of protein evolution derived from multiple protein families using a maximum-likelihood approach. *Mol. Biol. Evol.* **18**, 691–699 (2001).

75. G. Yu, D. K. Smith, H. Zhu, Y. Guan, T. T. Lam, ggtree : an r package for visualization and annotation of phylogenetic trees with their covariates and other associated data. *Methods Ecol. Evol.* **8**, 28–36 (2017).

76. J. Huerta-Cepas, *et al.*, eggNOG 4.5: a hierarchical orthology framework with improved functional annotations for eukaryotic, prokaryotic and viral sequences. *Nucleic Acids Res.* **44**, D286-93 (2016).

77. M. Kanehisa, S. Goto, KEGG: kyoto encyclopedia of genes and genomes. *Nucleic Acids Res.* **28**, 27–30 (2000).

78. I. Rivals, L. Personnaz, L. Taing, M.-C. Potier, Enrichment or depletion of a GO category within a class of genes: which test? *Bioinformatics* **23**, 401–407 (2007).

79. G. Yu, L.-G. Wang, Y. Han, Q.-Y. He, clusterProfiler: an R package for comparing biological themes among gene clusters. *OMICS* **16**, 284–287 (2012).

80. T. Hackl, *et al.*, Novel integrative elements and genomic plasticity in ocean ecosystems. *Cold Spring Harbor Laboratory*, 2020.12.28.424599 (2020).

81. S. Avrani, O. Wurtzel, I. Sharon, R. Sorek, D. Lindell, Genomic island variability facilitates Prochlorococcus-virus coexistence. *Nature* **474**, 604–608 (2011).

82. B. Buchfink, C. Xie, D. H. Huson, Fast and sensitive protein alignment using DIAMOND. *Nat. Methods* **12**, 59–60 (2015).

83. R. Schlitzer, *et al.*, The GEOTRACES Intermediate Data Product 2017. *Chem. Geol.* **493**, 210–223 (2018).

84. L. A. Salt, S. M. A. C. van Heuven, M. E. Claus, E. M. Jones, H. J. W. de Baar, Rapid acidification of mode and intermediate waters in the southwestern Atlantic Ocean. *Biogeosciences* **12**, 1387–1401 (2015).

85. M. J. A. Rijkenberg, *et al.*, The distribution of dissolved iron in the West Atlantic Ocean. *PLoS One* **9**, e101323 (2014).

86. N. J. Wyatt, *et al.*, Biogeochemical cycling of dissolved zinc along the GEOTRACES South Atlantic transect GA10 at 40°S: Dissolved zinc in the Atlantic at 40° S. *Global Biogeochem. Cycles* **28**, 44–56 (2014).

87. S. Speich, *et al.*, "Environmental context of all samples from the Tara Oceans Expedition (2009-2013), about the water column features at the sampling location" in (PANGAEA, 2017).

88. M. D. Ashkezari, *et al.*, Simons Collaborative Marine Atlas Project (Simons CMAP): an open-source portal to share, visualize and analyze ocean data. *bioRxiv*, 2021.02.16.431537 (2021).

89. L. Weinhold, *et al.*, A Random Forest Approach for Bounded Outcome Variables. *J. Comput. Graph. Stat.*, 1–20 (2019).

90. A. E. Teschendorff, Avoiding common pitfalls in machine learning omic data science. *Nat. Mater.* **18**, 422–427 (2019).

91. V. Zuber, K. Strimmer, High-Dimensional Regression and Variable Selection Using CAR Scores. *Stat. Appl. Genet. Mol. Biol.* **10** (2011).

92. M. Wright, A. Ziegler, ranger: A Fast Implementation of Random Forests for High Dimensional Data in C++ and R. *Journal of Statistical Software, Articles* **77**, 1–17 (2017).

93. M. Kursa, W. Rudnicki, Feature Selection with the Boruta Package. *Journal of Statistical Software, Articles* **36**, 1–13 (2010).

94. B. D. Martin, D. Witten, A. D. Willis, Modeling microbial abundances and dysbiosis with beta-binomial regression. *Ann. Appl. Stat.* **14**, 94–115 (2020).

95. S. N. Wood, Generalized Additive Models: An Introduction with R (2017).

96. L. R. Moore, *et al.*, Culturing the marine cyanobacterium Prochlorococcus. *Limnol. Oceanogr. Methods* **5**, 353–362 (2007).

97. J. J. Morris, R. Kirkegaard, M. J. Szul, Z. I. Johnson, E. R. Zinser, Facilitation of robust growth of Prochlorococcus colonies and dilute liquid cultures by “helper” heterotrophic bacteria. *Appl. Environ. Microbiol.* **74**, 4530–4534 (2008).

98. M. A. Saito, J. W. Moffett, S. W. Chisholm, J. B. Waterbury, Cobalt limitation and uptake in Prochlorococcus. *Limnol. Oceanogr.* **47**, 1629–1636 (2002).

99. E. R. Zinser, *et al.*, Prochlorococcus ecotype abundances in the North Atlantic Ocean as revealed by an improved quantitative PCR method. *Appl. Environ. Microbiol.* **72**, 723–732 (2006).

100. R. R. Malmstrom, *et al.*, Temporal dynamics of Prochlorococcus ecotypes in the Atlantic and Pacific oceans. *ISME J.* **4**, 1252–1264 (2010).

.101. L. D. Quin, A. J. Williams, Practical interpretation of P-31 NMR spectra and computer assisted structure verification. Technical Report: Advanced Chemistry Development, Toronto, CA, (2004).

102. A. Zambo, *et al.,* Nucleoside 2´,3´-cyclic monophosphates in Aphanizomenon flosaquae detected through nuclear magnetic resonance and mass spectrometry. *J. Ag Food Chemistry***. 67**, 12780-12785 (2019).

103. B. M. Fontaine, *et al.*, RNase I regulates Escherichia coli 2’,3’-cyclic nucleotide monophosphate levels and biofilm formation. *Biochem. J* **475**, 1491–1506 (2018).

104. Y. Sokurenko, Zelenikhin P., Ulyanova V., Kolpakov A., Muller D., Ilinskaya O, Identification of 2′,3′-cGMP as an intermediate of RNA catalytic cleavage by binase and evaluation of its biological action. *Rus. J. Bioorg. Chem*. **41**, 31-36 (2015).

105. K. Blin, *et al.*, antiSMASH 5.0: updates to the secondary metabolite genome mining pipeline. *Nucleic Acids Res.* **47**, W81–W87 (2019).

106. J. Huerta-Cepas, *et al.*, Fast Genome-Wide Functional Annotation through Orthology Assignment by eggNOG-Mapper. *Mol. Biol. Evol.* **34**, 2115–2122 (2017).

Supplemental Dataset Legends

SI Dataset 1
Phosphonenolpyruvate mutase (PepM) sequence identifiers, parent genomes, HMM annotation, catalytic residues, taxonomic identification, and genome assembly source from GORG-Tropics and MARMICRODB.

SI Dataset 2
Methylphosphonate synthase (MpnS) family sequence identifiers, parent genomes, HMM annotation, catalytic residues, taxonomic identification, and genome assembly source from GORG-Tropics and MARMICRODB.

SI Dataset 3
Genomes from GORG-Tropics and MARMICRODB containing both phosphonate biosynthesis and catabolism genes.
